# Supplementary figures and images for: Toxic Y chromosome: Increased repeat expression and age-associated heterochromatin loss in male Drosophila with a young Y chromosome
Source: PLoS Genet. 2021 Apr 22;17(4):e1009438. doi: 10.1371/journal.pgen.1009438 (PMC8061872; doi:10.1371/journal.pgen.1009438)

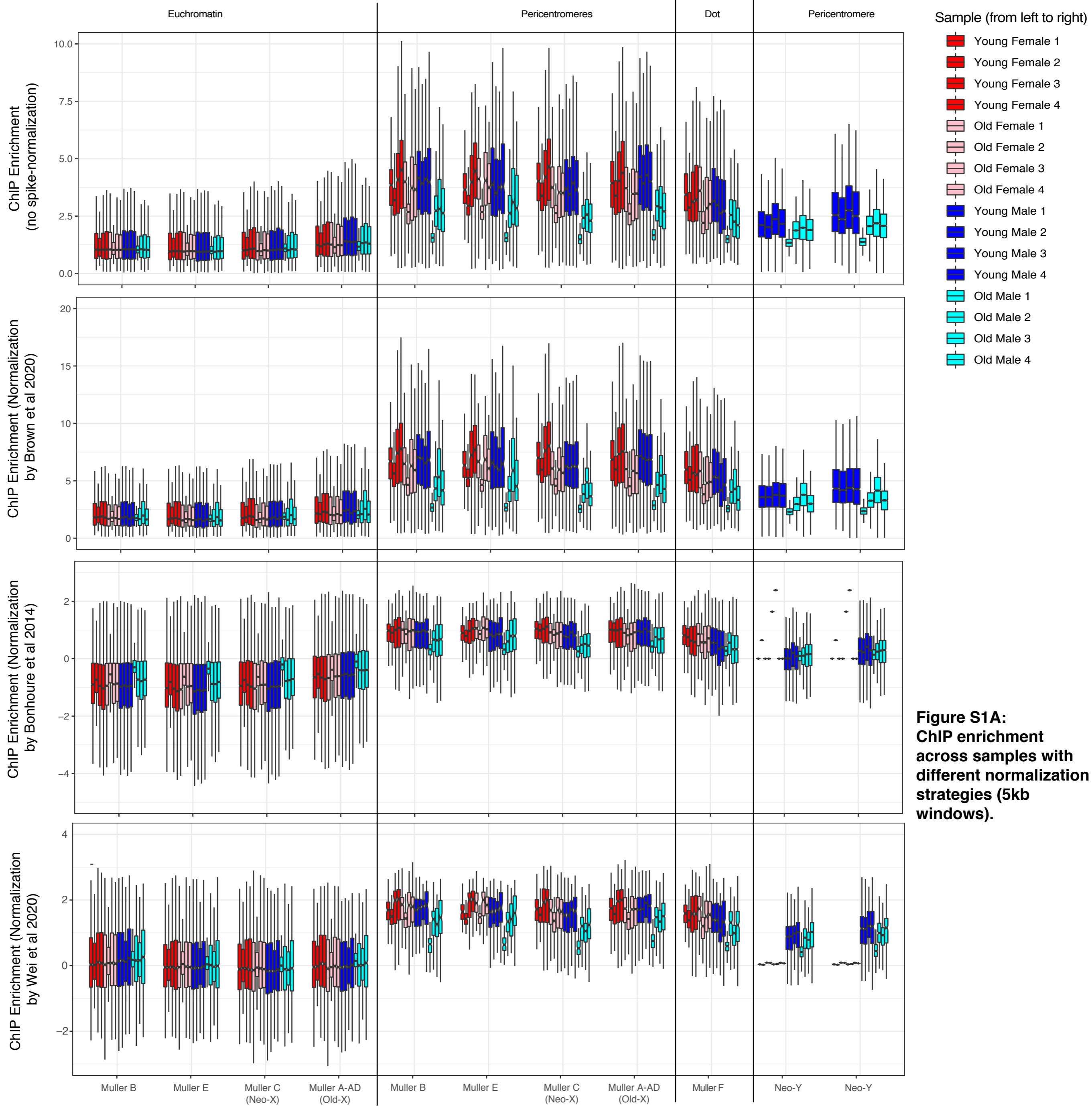

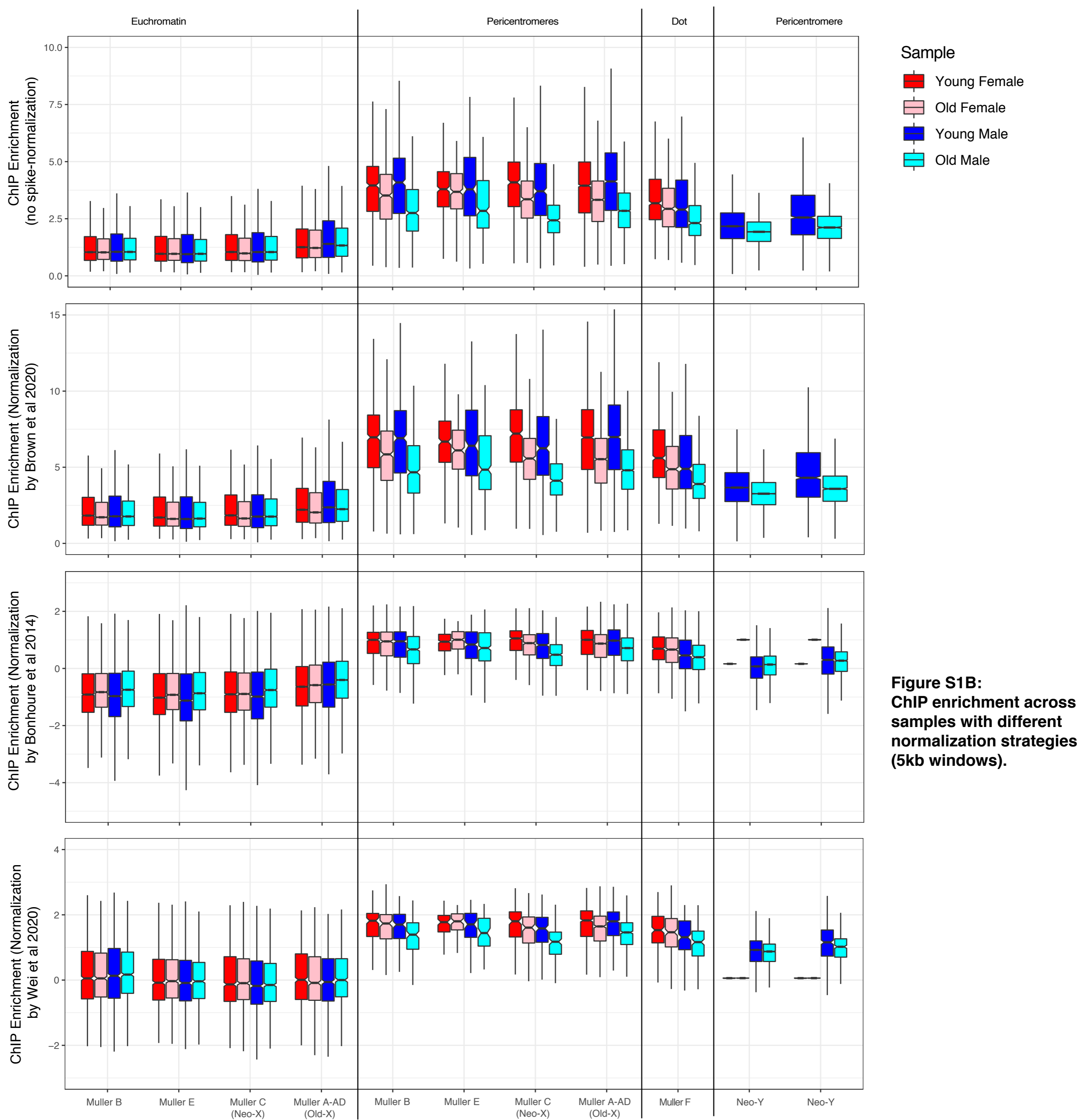

Supplement: S1 Fig — From top to bottom: No spike in normalization: ChIP enrichment normalized by coverage of the autosomes for all biological samples and replicates, ignoring the spike-in. Brown et al. (2020) normalization: ChIP enrichment normalized by assessing proportion of reads derived from sample and spike-in both the ChIP and Input. Bonhoure et al. (2014) normalization: ChIP enrichment normalized by employing a regression-based method using the D. melanogaster spike-in. Wei et al. (2020) normalization: ChIP enrichment normalized by employing a quantile-quantile method using the D. melanogaster spike-in. A. Enrichment for each sample replicate. B. Average enrichment across samples. We omitted the first Old Male sample for all averaged Old Male values because it had lower enrichment signal compared to other samples in both the sample and the spike-in, indicative of inefficient ChIP pull-down. (PDF) [file pgen.1009438.s001.pdf]

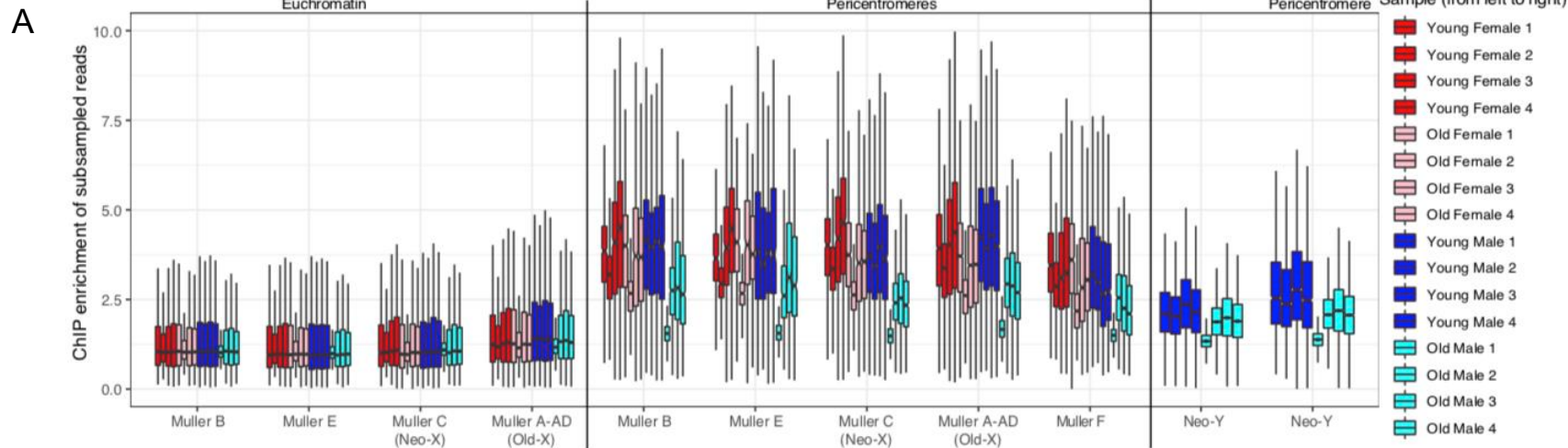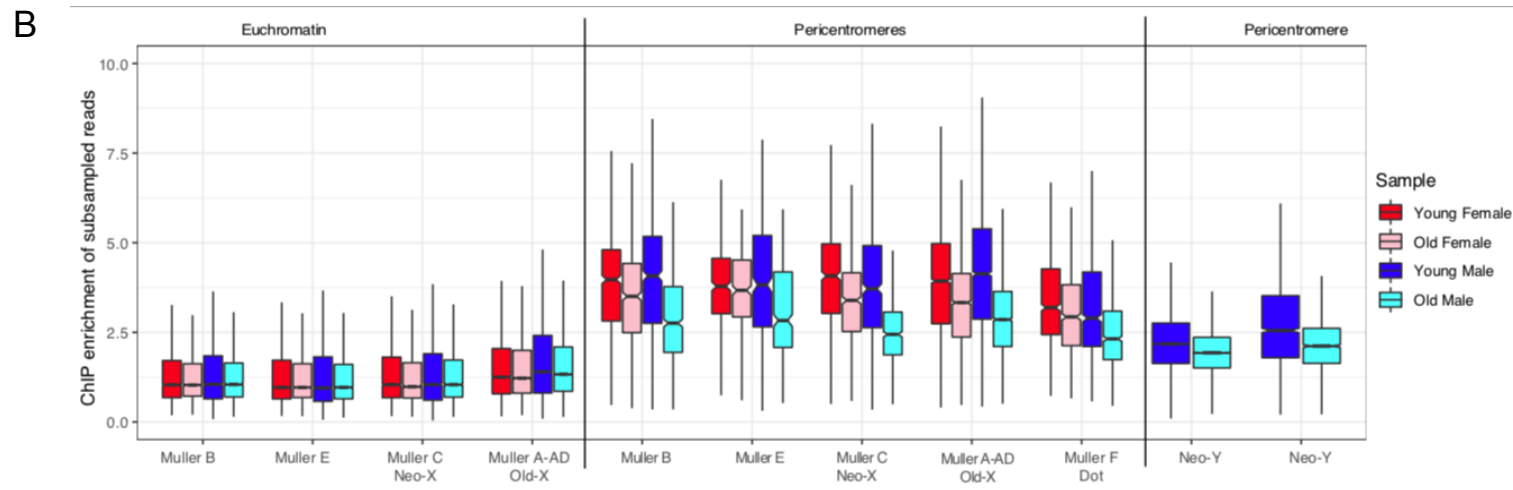

**Figure S2: ChIP signal after subsampling reads to sample with smallest number of reads**

Supplement: S2 Fig — A. Enrichment for each sample replicate. B. Average enrichment across samples. Enrichment is normalized without spike-in and omits the failed Old Male replicate. (PDF) [file pgen.1009438.s002.pdf]

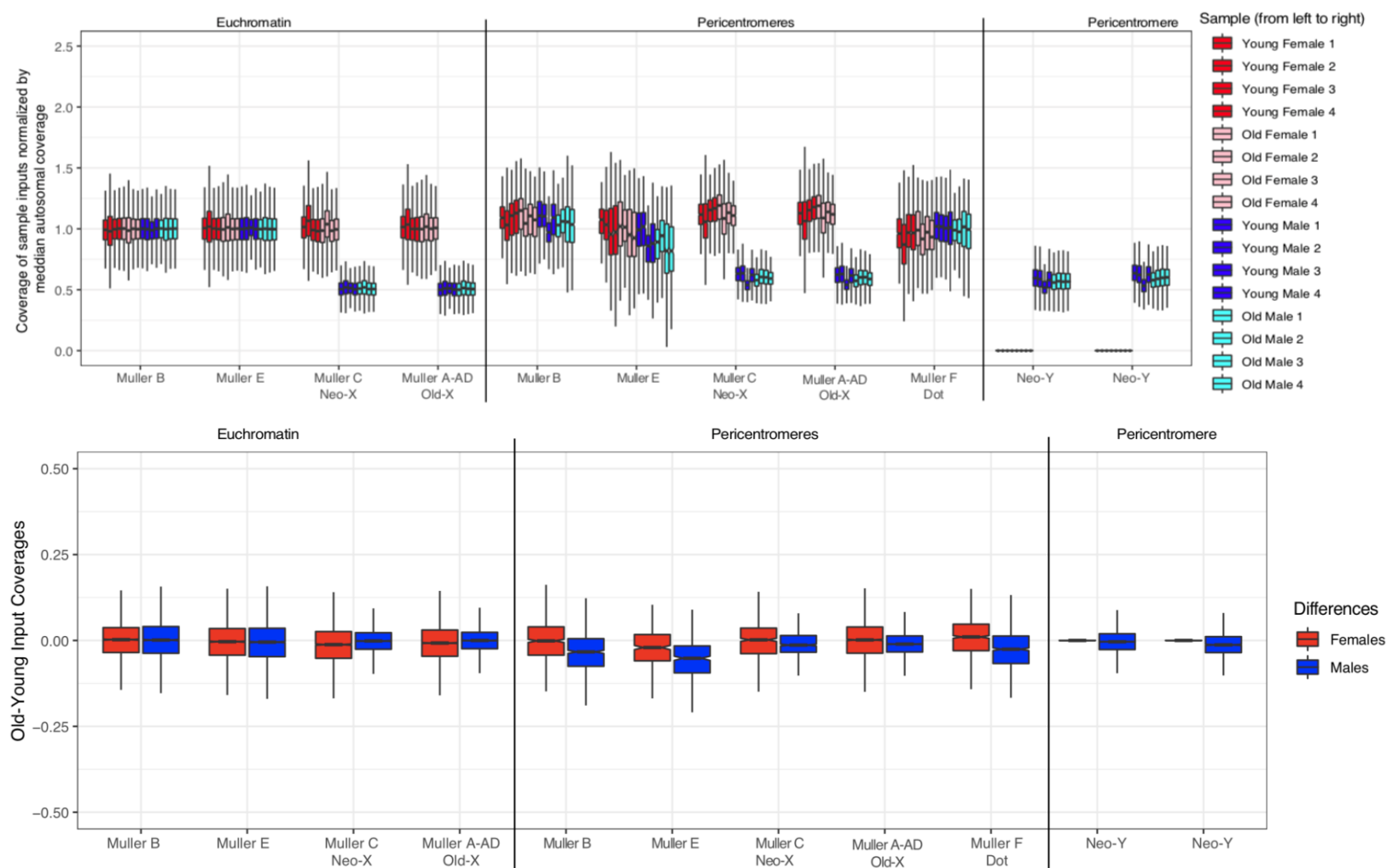

**Figure S4: Input coverage across biological samples and differences between ages**

Supplement: S4 Fig — (Top) Coverage of the input DNA across samples normalized by the median autosomal coverage. (Bottom) Mean coverage of the input DNA across samples. (PDF) [file pgen.1009438.s004.pdf]

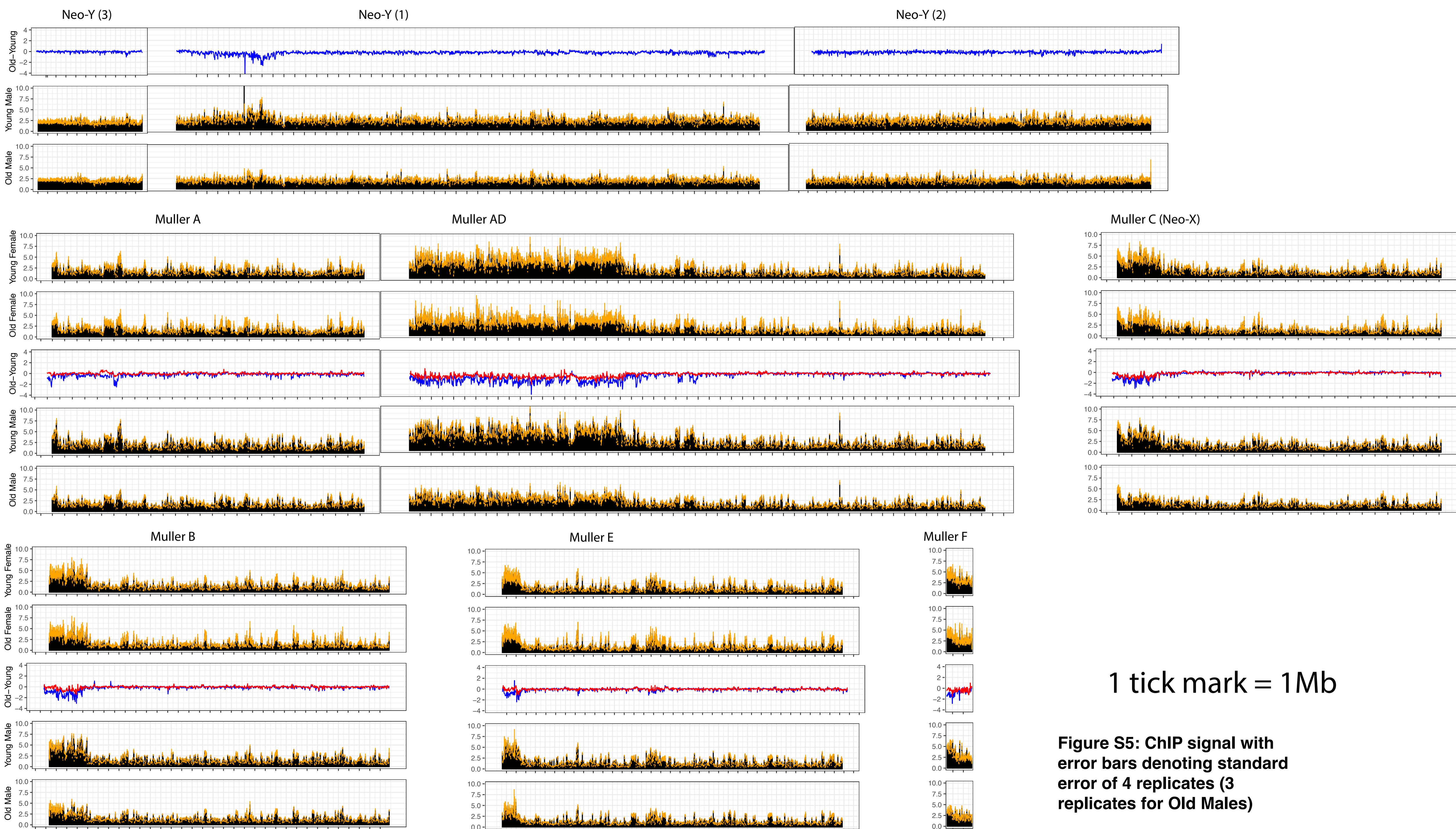

Supplement: S5 Fig — 1 tick mark = 1Mb on the x-axis. (PDF) [file pgen.1009438.s005.pdf]

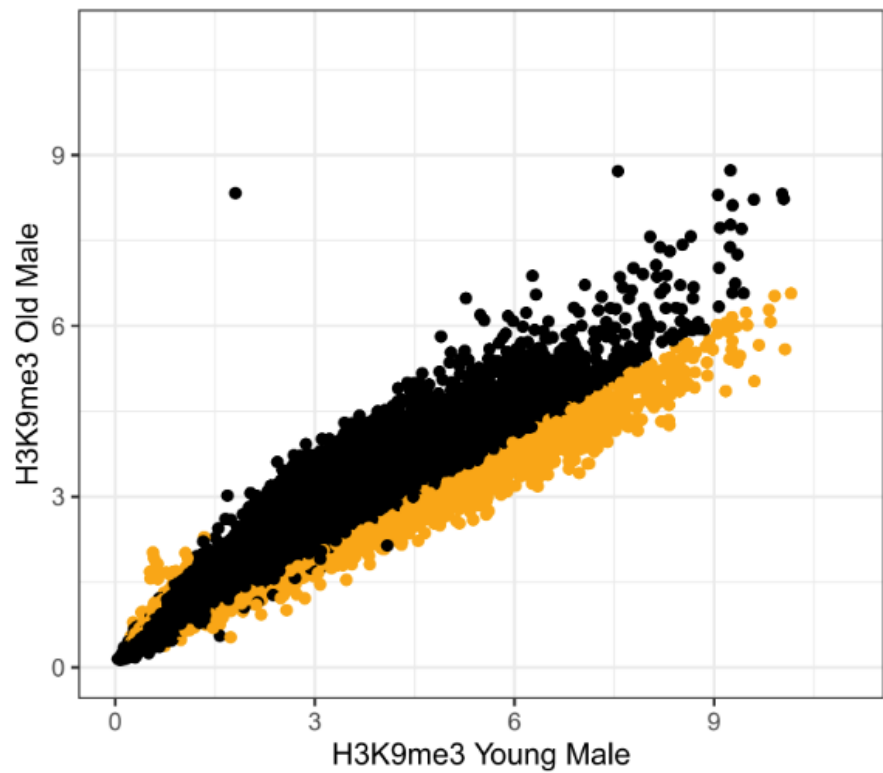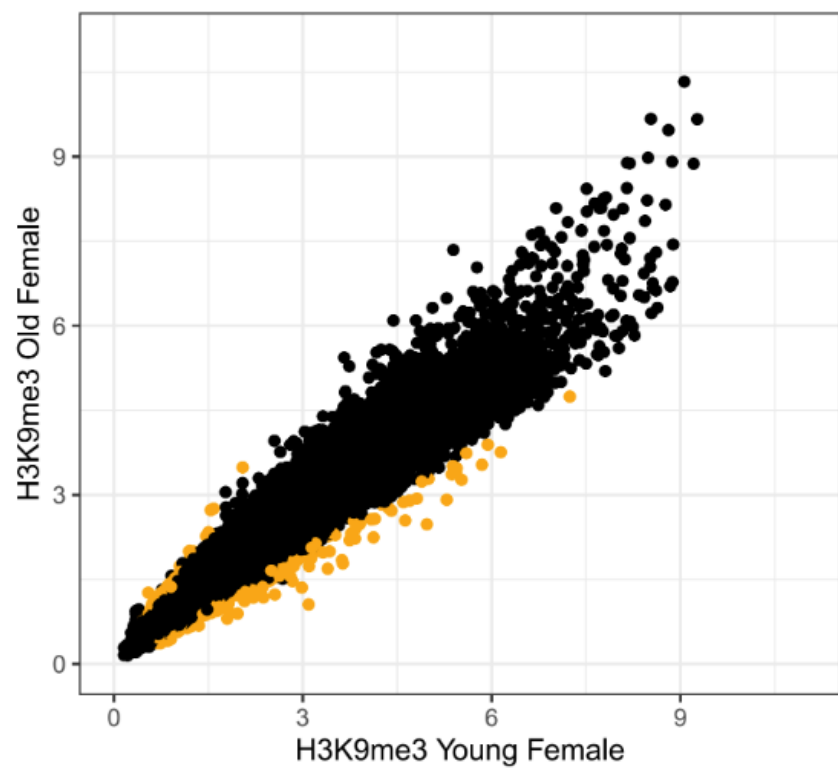

**Figure S6: Scatterplots of ChIP enrichment within sexes**

Supplement: S6 Fig — Each data point represents enrichment of old/young male (left) or female (right) samples at a 5---kb window. Data highlighted in yellow denote windows with 50% higher/lower enrichment between samples (p < 0.05, two-tailed Student’s t-test). (PDF) [file pgen.1009438.s006.pdf]

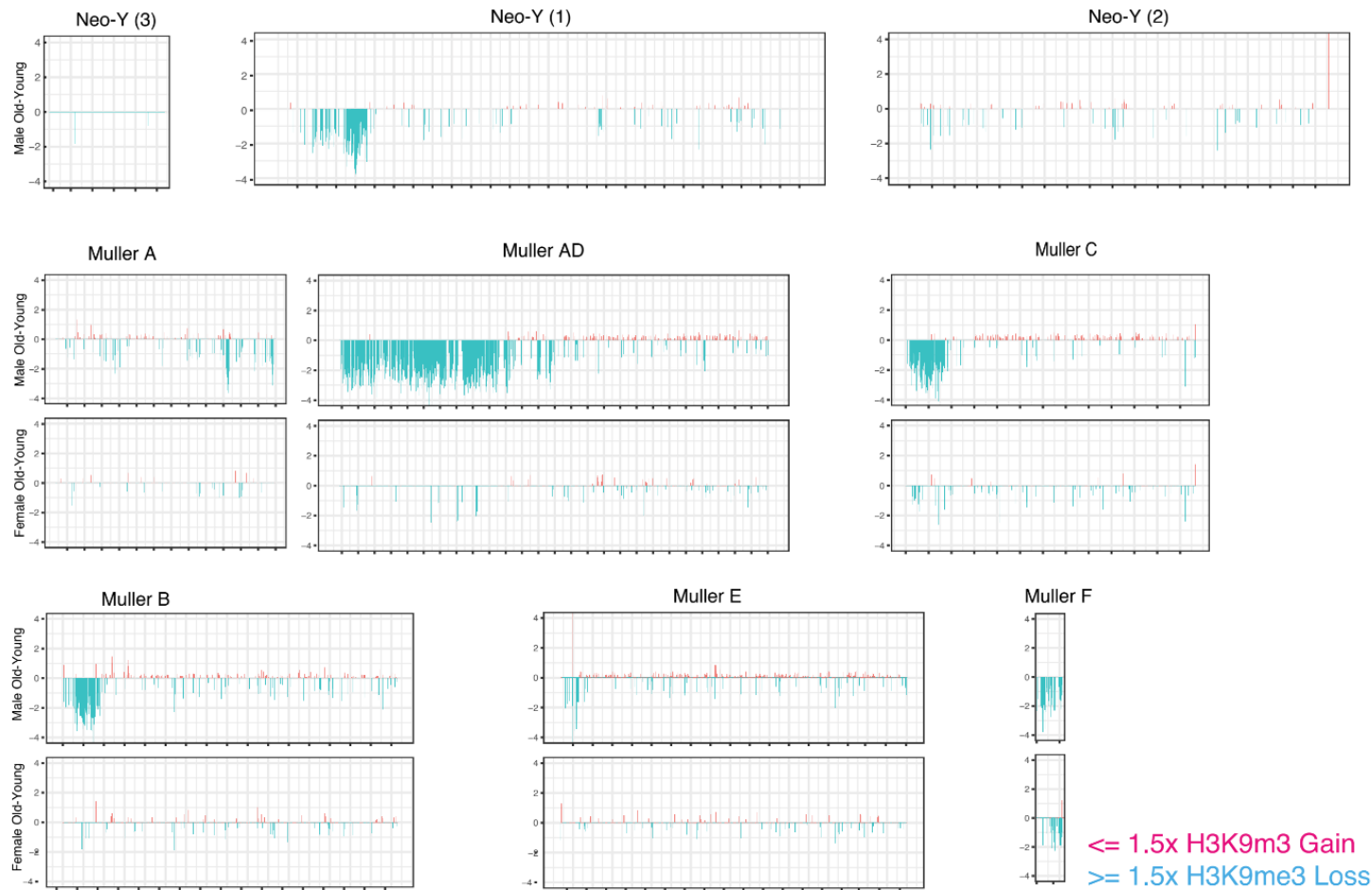

**Figure S8: Regions with at least 1.5x H3K9me3 up-/down-regulation within sex over aging (5kb windows).**

Supplement: S8 Fig — males and B. females. Boundaries are indicated by darker region on the chromosome diagrams of the major chromosome arms. Subtraction plots show higher H3K9me2 signal in young (blue) or old (red) flies. Data represents the mean of 4 biological replicates (3 for old males; in black) or the difference of means (subtraction plots, blue or red). (PDF) [file pgen.1009438.s008.pdf]

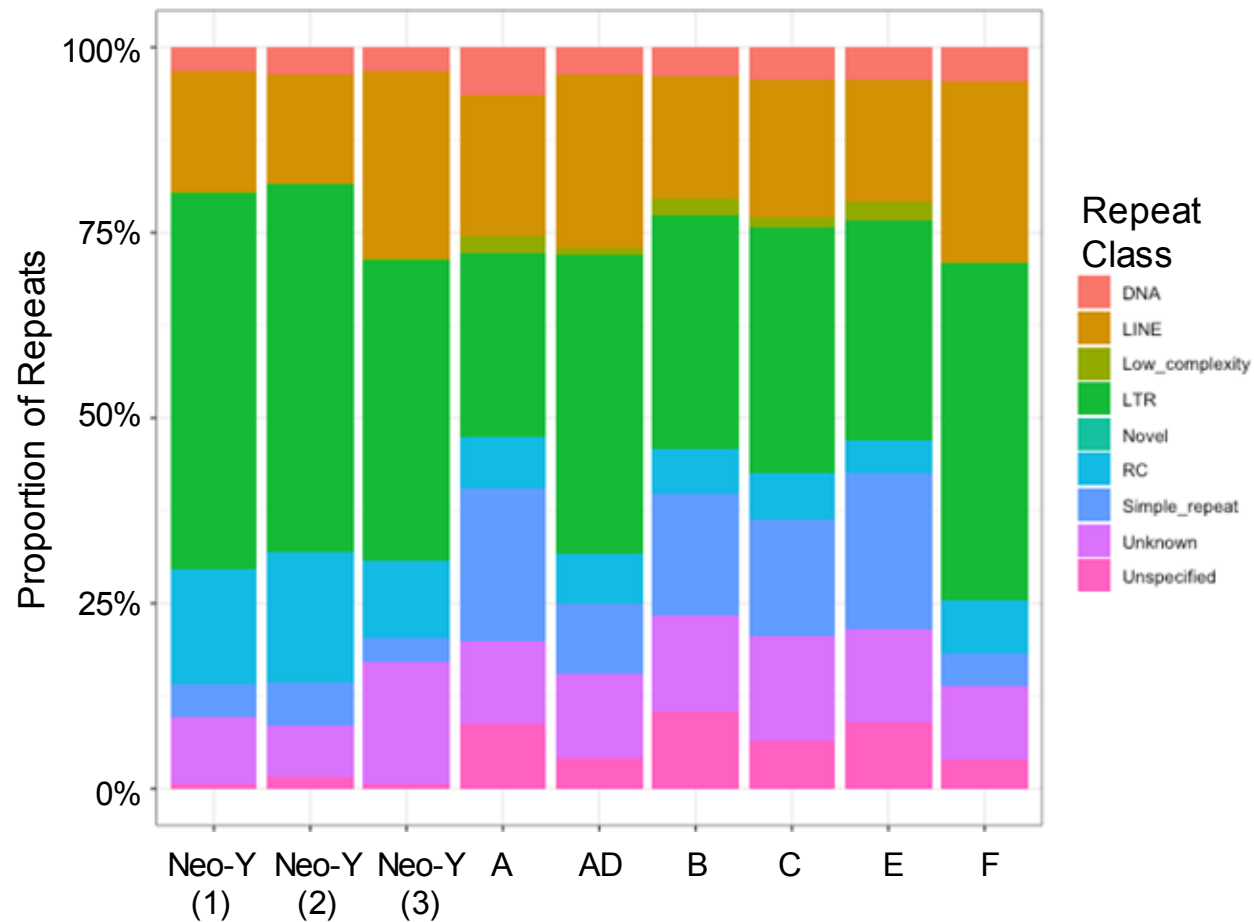

**Figure S11: Repeat composition on a per chromosome basis.**

Supplement: S11 Fig — Plotted for each Muller element is the total length of repeats (bp) divided by the entire chromosome length (bp). (PDF) [file pgen.1009438.s011.pdf]

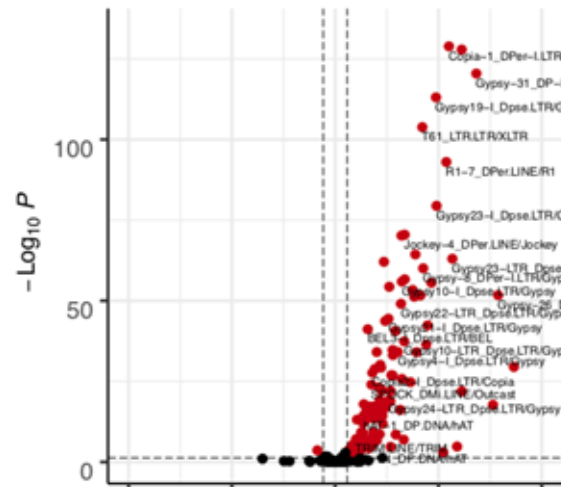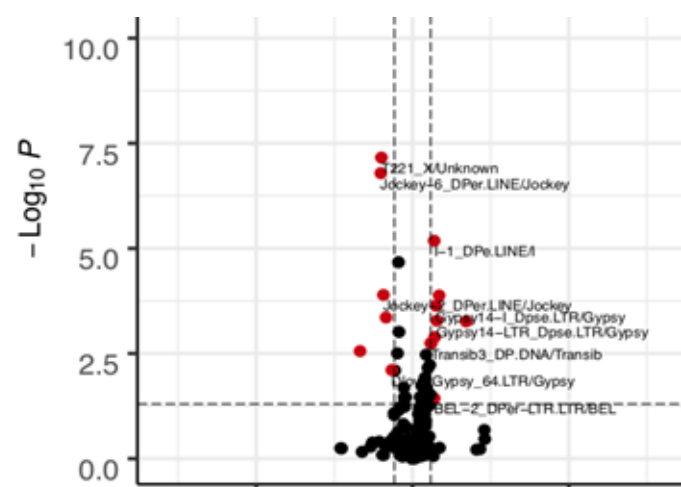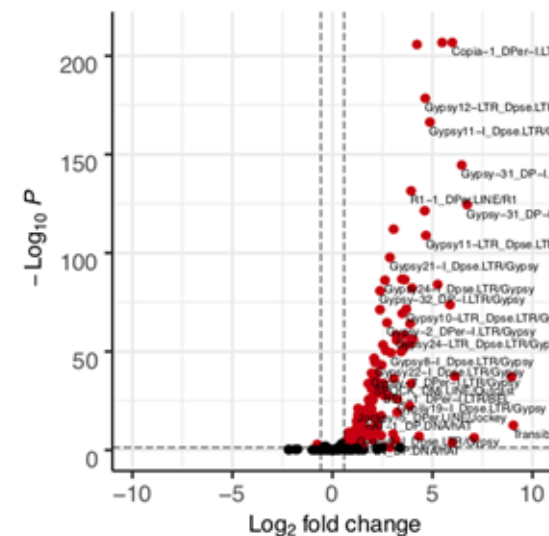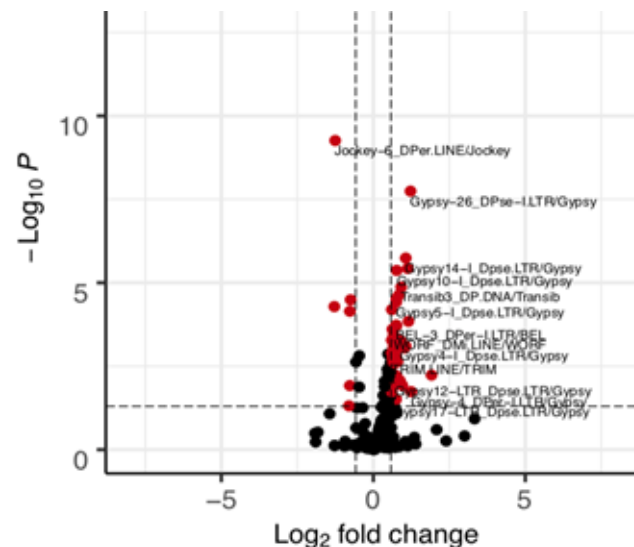

Figure S13: Volcano plots for TE expression plots by sex and age.

Supplement: S13 Fig — Left: (Top) TEs in young females v. young males, (bottom) TEs in old females vs old males. Fold change is indicative of male/female. Right: (Top) TEs in young males vs old males, (bottom) TEs in young females vs old females. Fold change is indicative of young/old. (PDF) [file pgen.1009438.s013.pdf]

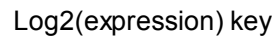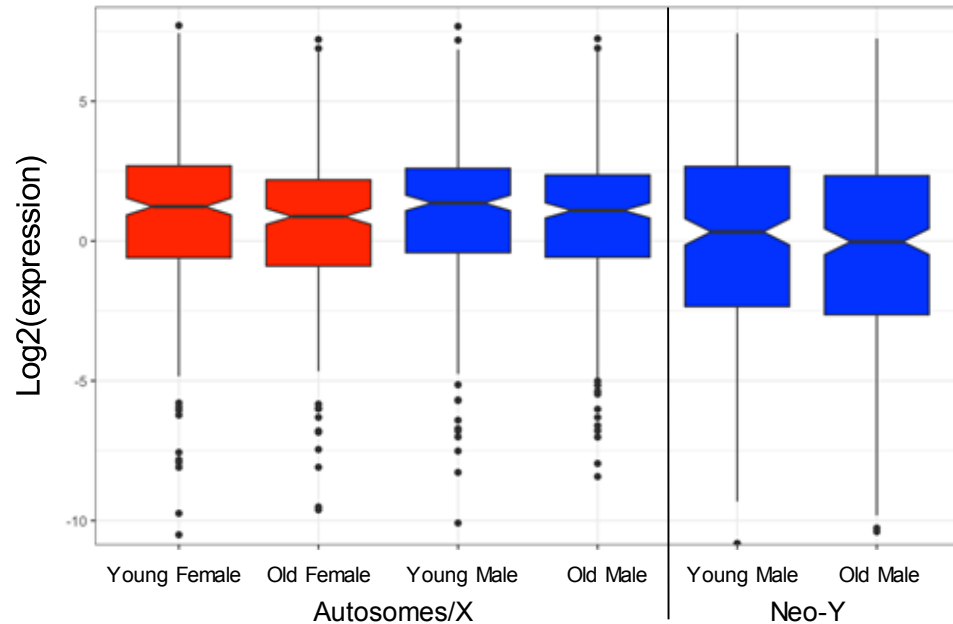

**Figure S14: TE expression by sex/age and chromosome group.**

Supplement: S14 Fig — (PDF) [file pgen.1009438.s014.pdf]

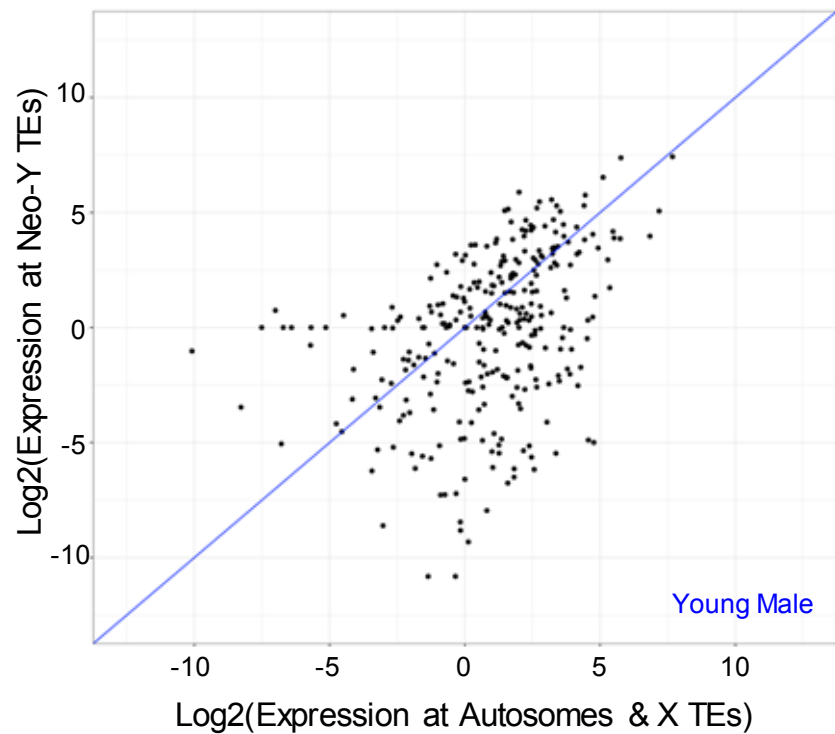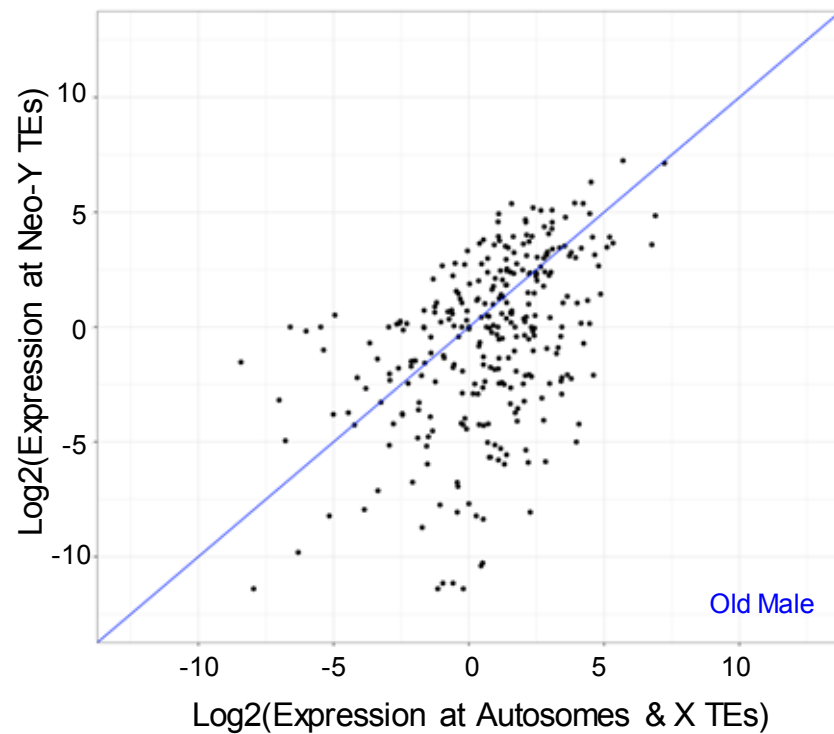

**Figure S15: Male TE expression from the Autosomes/X and the Neo-Y.**

Supplement: S15 Fig — Diagonal line denotes 1:1 equal expression for TEs from both chromosome groups. (PDF) [file pgen.1009438.s015.pdf]

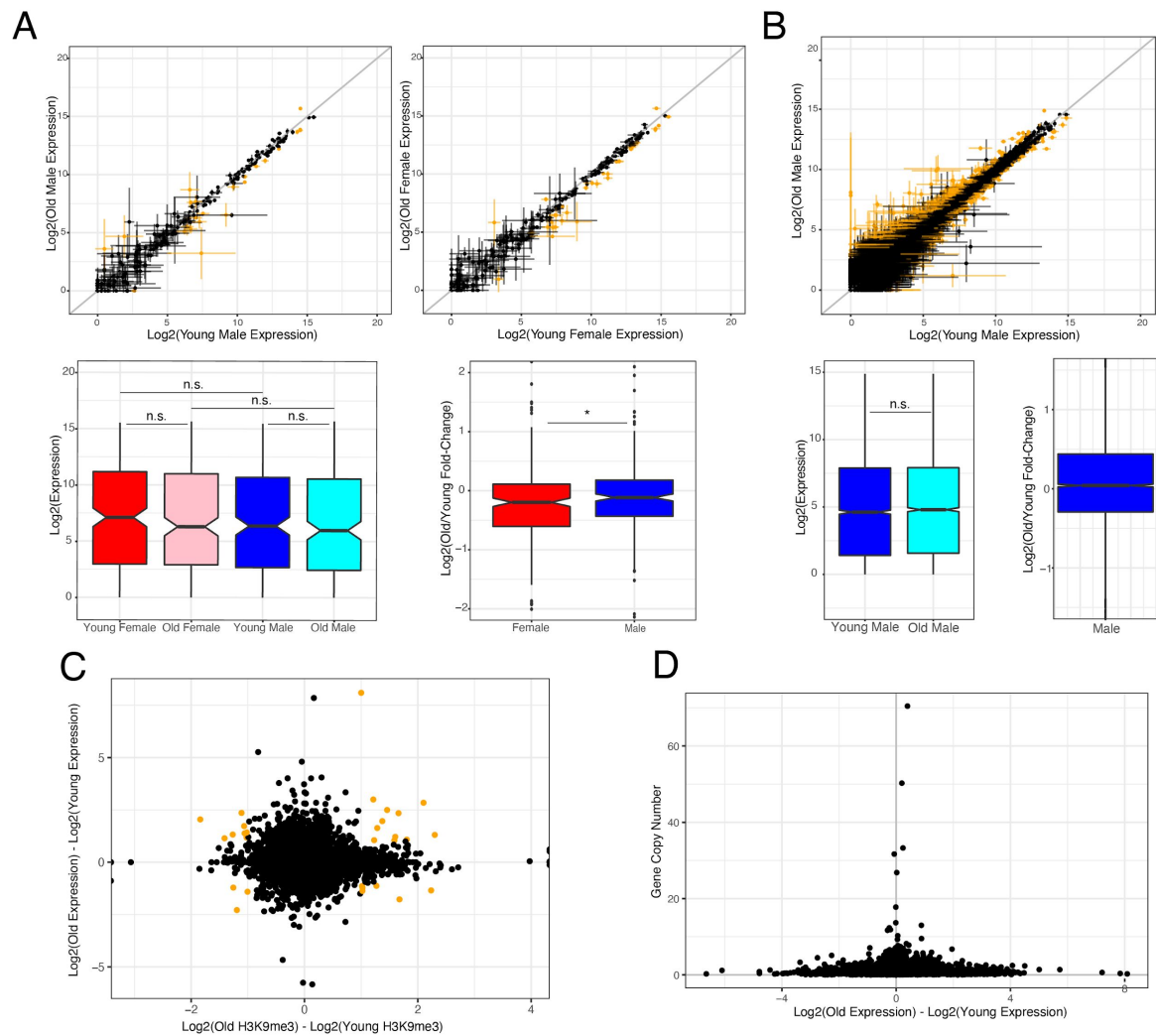

Figure S18: Heterochromatin and expression of pericentromeric and Neo-Y genes.

Supplement: S18 Fig — A. Log2 expression of genes located in the pericentromeres within sexes. Data represents mean values of 3 replicates with bars denoting standard error. Data highlighted in yellow denotes genes with significantly 50% higher or lower expression when comparing fold-change between samples (Wald test, p < 0.05). Grey line indicates 1:1 expression between samples. Boxplot values represent mean values of 3 replicates with significance values calculated using the Wilcoxon test (* p< 0.05, ** p<0.01, *** p<1e-5). B. Log2 expression of genes located on the neo-Y between young and old males. Data represented in a similar manner as in A and data highlighted shows genes that are up-regulated (top-left) and/or down-regulated (bottom-right) by 50% during aging (Wald test, p < 0.05). C. Age-related differences in log2 H3K9me3 enrichment and log2 expression of neo-Y genes. Each data represents the difference of mean values between young and old males. Color-code indicates differentially expressed/enriched comparisons: black: absolute log2(fold-change) < 1in both H3K9me3 and expression; yellow: absolute log2(fold-change)> = 1 in both H3K9me3 and expression. D. Log2 expression differences in genes on the neo-Y and their respective gene copy number. Values denote the difference of mean values between young and old males. Grey line denotes 0 expression difference between the ages. (PDF) [file pgen.1009438.s018.pdf]
