# Supplementary material for: Toxic Y chromosome: Increased repeat expression and age-associated heterochromatin loss in male Drosophila with a young Y chromosome
Source: PLoS Genet. 2021 Apr 22;17(4):e1009438. doi: 10.1371/journal.pgen.1009438 (PMC8061872; doi:10.1371/journal.pgen.1009438)
Supplement: S3 Fig — (PDF) [file pgen.1009438.s003.pdf]

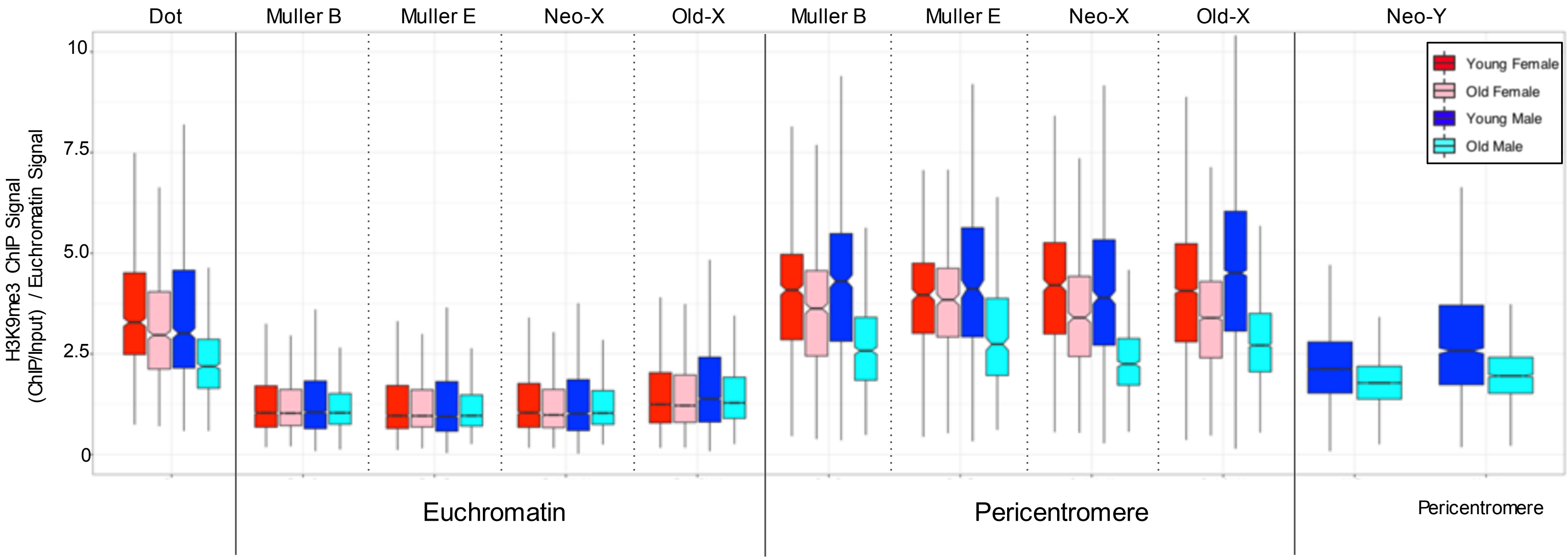

Figure S3: ChIP enrichment across samples with uniquely mapped reads (5kb windows). We used MAPQ > 3 as a cutoff for uniquely mapped reads.
