# Supplementary material for: Toxic Y chromosome: Increased repeat expression and age-associated heterochromatin loss in male Drosophila with a young Y chromosome
Source: PLoS Genet. 2021 Apr 22;17(4):e1009438. doi: 10.1371/journal.pgen.1009438 (PMC8061872; doi:10.1371/journal.pgen.1009438)
Supplement: S7 Fig — Data values represent the difference of means from old and young samples. One tick mark on the x-axis is equal to 2Mb. (PDF) [file pgen.1009438.s007.pdf]

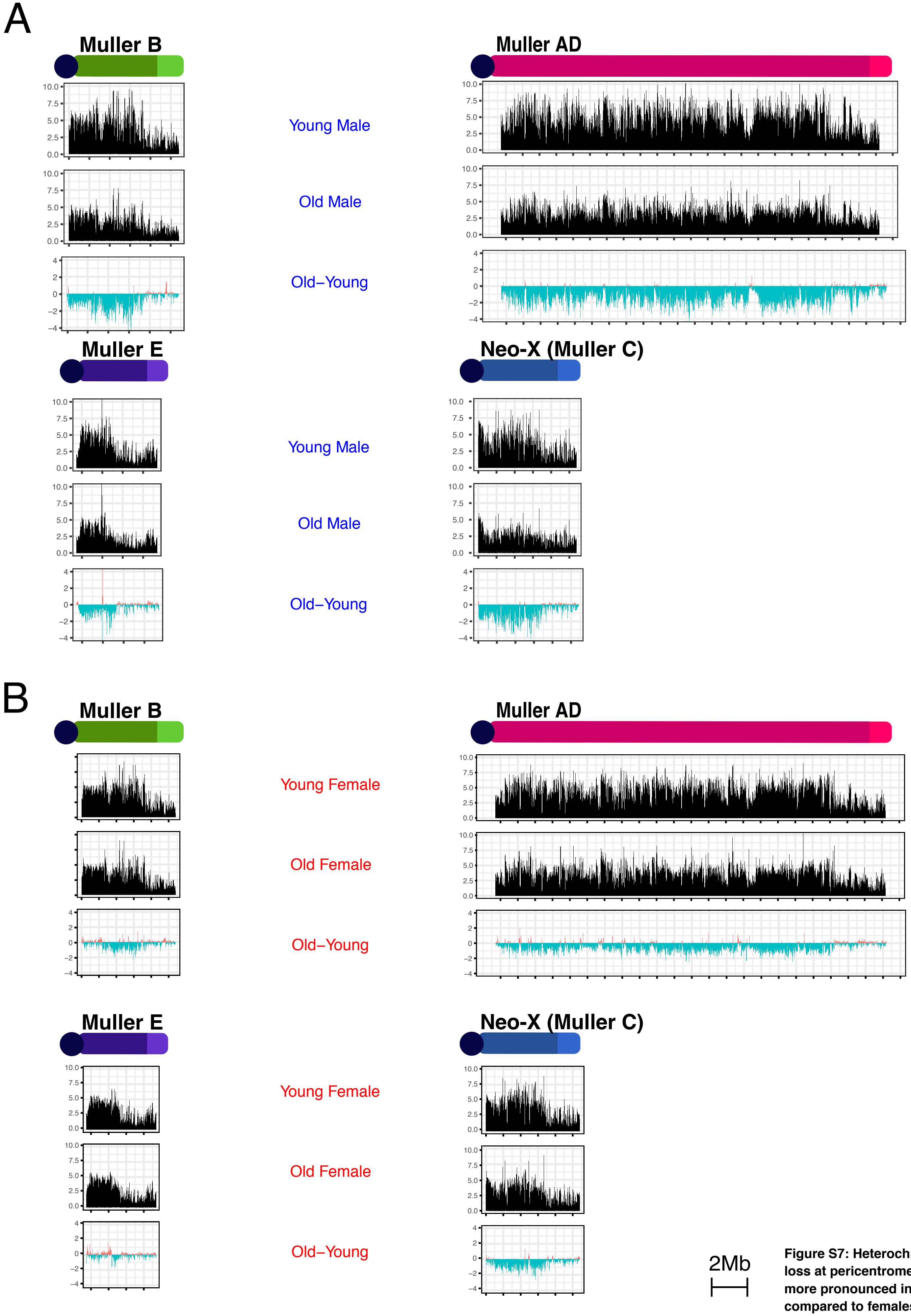

Figure S7: Heterochromatin loss at pericentromeres is more pronounced in males compared to females.
