# Supplementary material for: Toxic Y chromosome: Increased repeat expression and age-associated heterochromatin loss in male Drosophila with a young Y chromosome
Source: PLoS Genet. 2021 Apr 22;17(4):e1009438. doi: 10.1371/journal.pgen.1009438 (PMC8061872; doi:10.1371/journal.pgen.1009438)
Supplement: S9 Fig — (PDF) [file pgen.1009438.s009.pdf]

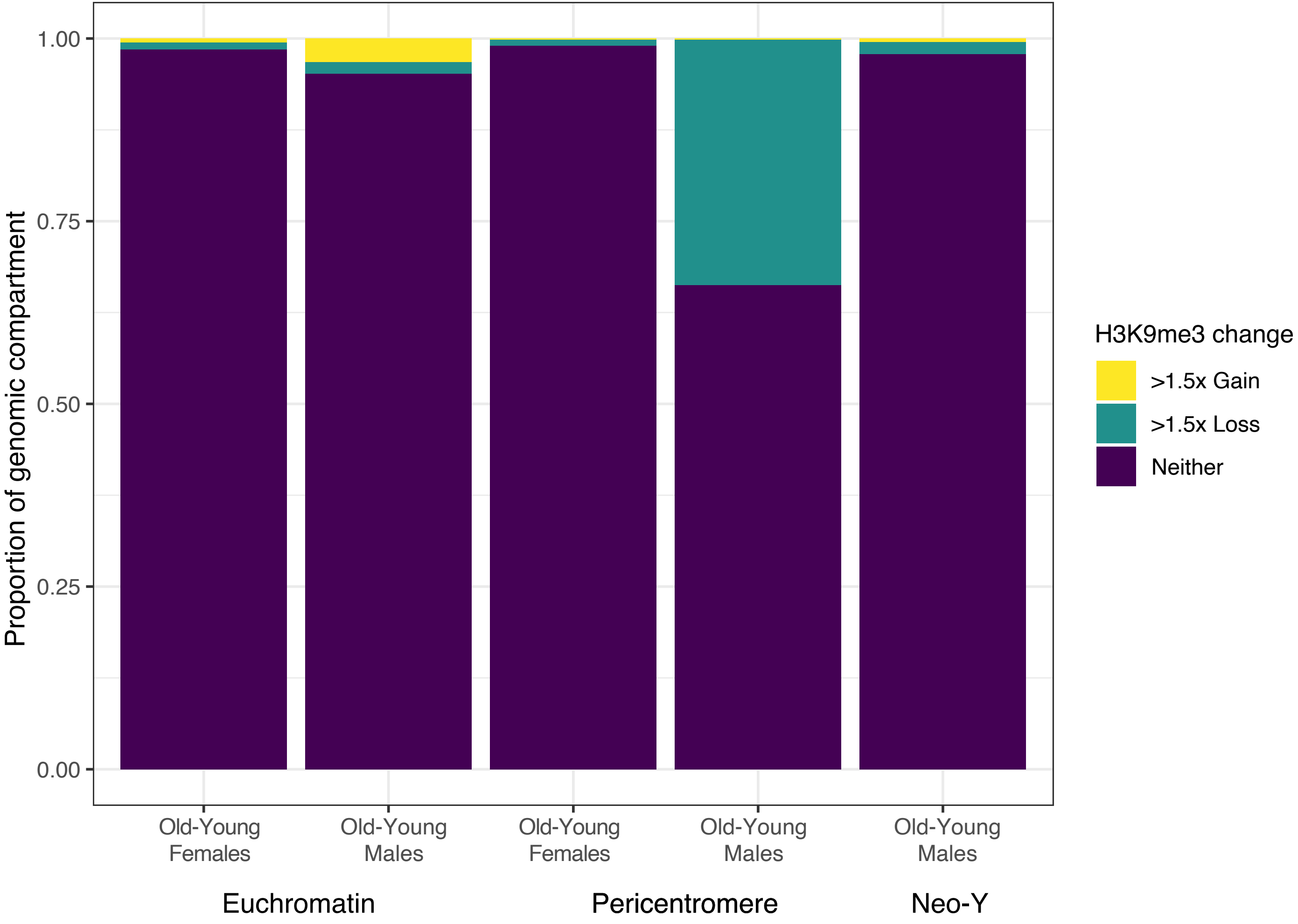

Figure S9: Sex-specific H3K9me3 gain/loss by compartment (euchromatin/heterochromatin/neo-Y) over aging.
