# Supplementary material for: Toxic Y chromosome: Increased repeat expression and age-associated heterochromatin loss in male Drosophila with a young Y chromosome
Source: PLoS Genet. 2021 Apr 22;17(4):e1009438. doi: 10.1371/journal.pgen.1009438 (PMC8061872; doi:10.1371/journal.pgen.1009438)
Supplement: S10 Fig — For all figures, arrowhead denotes neo-Y, arrows denote MullerA-AD, DAPI/DNA is blue channel, H3K9me3 is orange channel, and scale bar is 50μm. (PDF) [file pgen.1009438.s010.pdf]

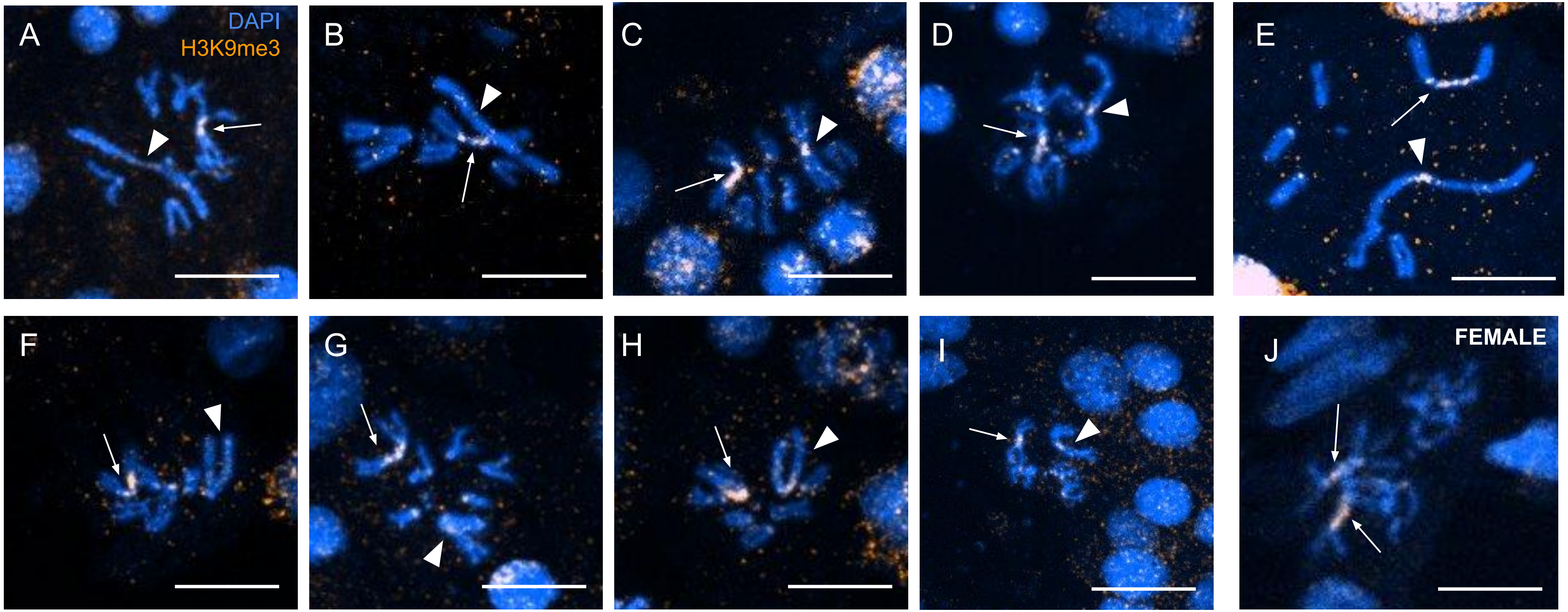

Figure S10: Immunofluorescence staining for H3K9me3 in males (A-I, replicates from different slides) and females (J). For all figures, arrowhead denotes neo-Y, arrows denote MullerA-AD, DAPI/DNA is blue channel, H3K9me3 is orange channel, and scale bar is 50μm
