# Supplementary material for: Toxic Y chromosome: Increased repeat expression and age-associated heterochromatin loss in male Drosophila with a young Y chromosome
Source: PLoS Genet. 2021 Apr 22;17(4):e1009438. doi: 10.1371/journal.pgen.1009438 (PMC8061872; doi:10.1371/journal.pgen.1009438)
Supplement: S12 Fig — Values represent the difference of means between young and old samples, grouped by chromosome and binned by amount of repeats (%) per 5kb-window. (PDF) [file pgen.1009438.s012.pdf]

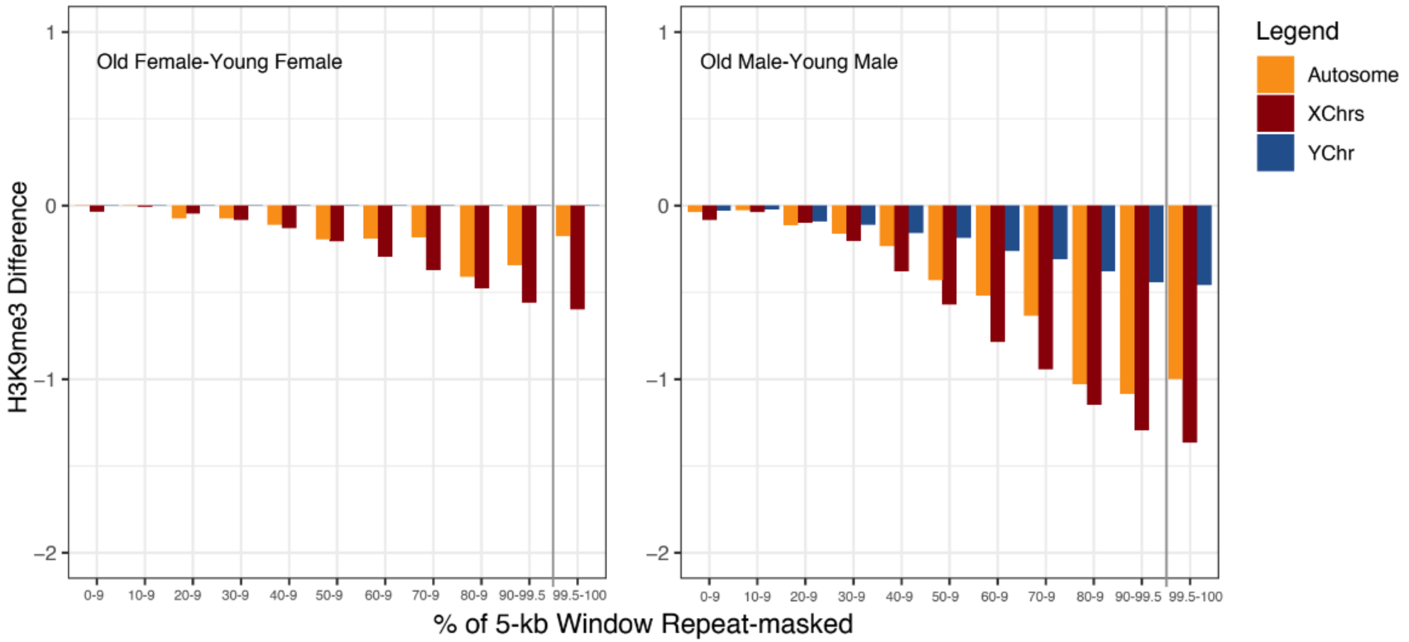

Figure S12: Differences in ChIP enrichment by binned repetitive content on a per chromosome basis (5kb windows).
