# Supplementary material for: Toxic Y chromosome: Increased repeat expression and age-associated heterochromatin loss in male Drosophila with a young Y chromosome
Source: PLoS Genet. 2021 Apr 22;17(4):e1009438. doi: 10.1371/journal.pgen.1009438 (PMC8061872; doi:10.1371/journal.pgen.1009438)
Supplement: S16 Fig — A-C. Shown are expression values for each biological sample accounting for TE copy number differences in the males and females when copies are calculated from A. adult gDNA, B. embryo gDNA, and C. genome repeat annotation. Expression values for each TE were normalized by their respective copy number in the male or female genome. Data plotted represents the averaged values of old and young female and male samples (3 replicates). Significance values calculated (n.s. not significant, * p<0.05, Wilcoxon test). D-F. Shown are Log2(Male/Female Expression) values for each TE along with the corresponding Log2(Male/Female DNA) values calculated from D. adult gDNA, E. embryo gDNA, and F. genome repeat annotation. Line indicates male/female expression is proportional to the male/female copy number of a specific TE. (PDF) [file pgen.1009438.s016.pdf]

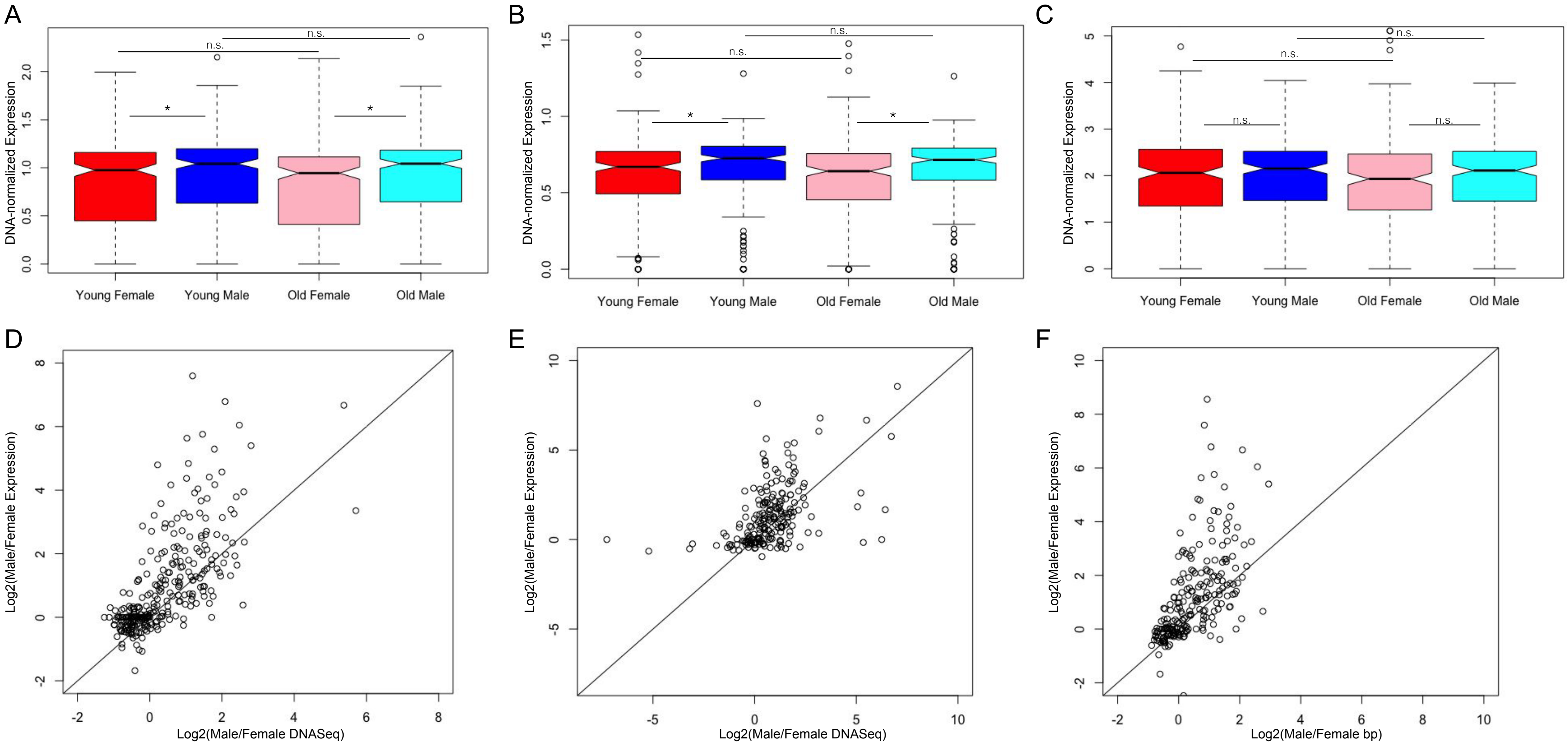


**Figure S16: Sex-specific differences in TE expression normalized by sex-specific differences in DNA copy number by (A) adult gDNA-Seq, (B) embryo gDNA-Seq, and (C) genome repeat annotation. Log2 male/female in RNA and DNA where copy number is from (D) adult gDNA-Seq, (E) embryo gDNA-Seq, (F) genome repeat annotation.**
