# Supplementary material for: Toxic Y chromosome: Increased repeat expression and age-associated heterochromatin loss in male Drosophila with a young Y chromosome
Source: PLoS Genet. 2021 Apr 22;17(4):e1009438. doi: 10.1371/journal.pgen.1009438 (PMC8061872; doi:10.1371/journal.pgen.1009438)
Supplement: S19 Fig — Heterochromatin cutoffs (dashed orange line) by % repeats overlap with regions of elevated H3K9me3 enrichment on (A) Muller B, (B) Muller E, (C) Muller C, and (D) Muller AD. (PDF) [file pgen.1009438.s019.pdf]

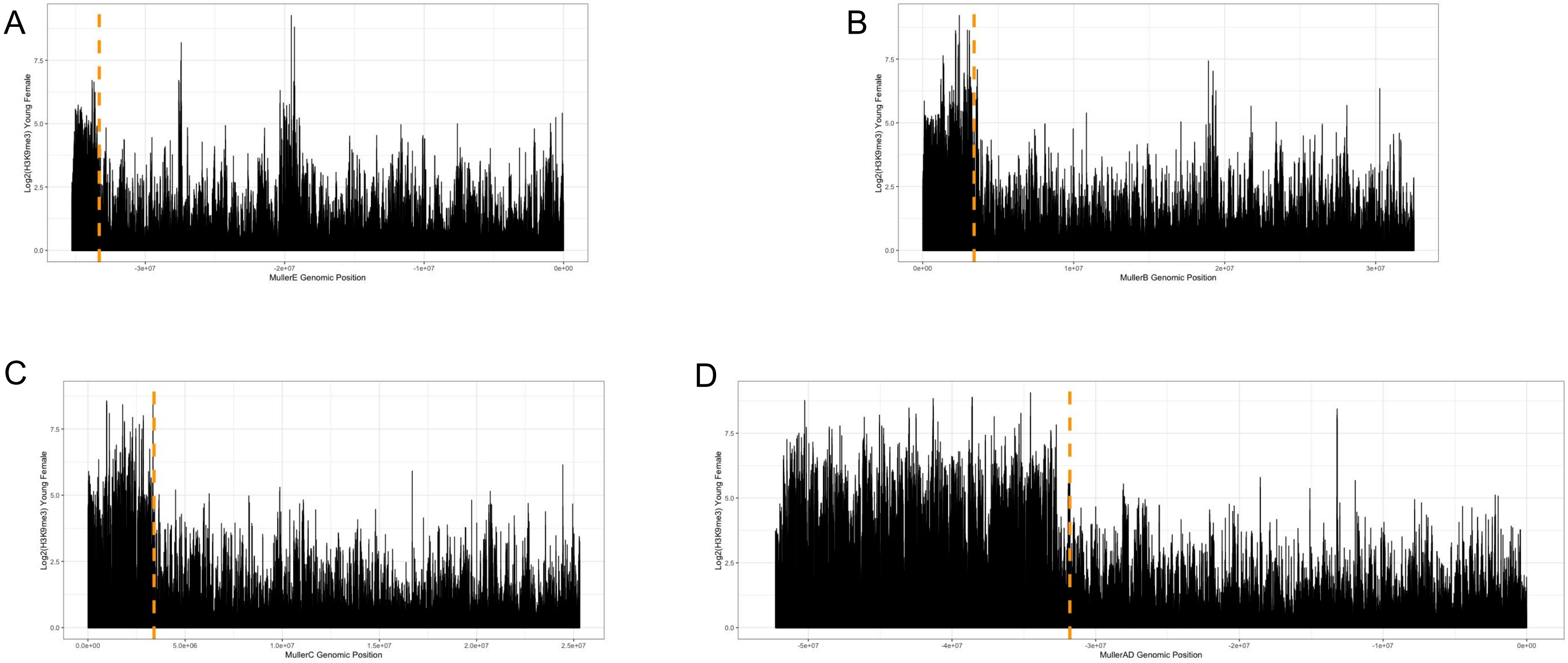

Figure S19: Heterochromatin cutoffs (dashed orange line) by % repeats overlap with regions of elevated H3K9me3 enrichment on (A) Muller B, (B) Muller E, (C) Muller C, and (D) Muller AD.
