# Supplementary material for: Toxic Y chromosome: Increased repeat expression and age-associated heterochromatin loss in male Drosophila with a young Y chromosome
Source: PLoS Genet. 2021 Apr 22;17(4):e1009438. doi: 10.1371/journal.pgen.1009438 (PMC8061872; doi:10.1371/journal.pgen.1009438)
Supplement: S1 Table — Description of samples/tissues used for 4 rounds/batches of ChIP-seq experiments. (PDF) [file pgen.1009438.s020.pdf]

**Table S1 - Overview of ChIP data generated**

Description of samples/tissues used for 4 rounds/batches of ChIP-seq experiments.

| Sex | Age | Batch | Data type | Antibody | reads | Spike-in | Tissue | Figure | SRA Accession |
|---|---|---|---|---|---|---|---|---|---|
| Female | 5 day | 1 | ChIP-Seq | H3K9me3 | 100 bp PE | *D. melanogaster* | brain | Fig. 2-3, 5 | SRR12192013 |
| Female | 5 day | 2 | ChIP-Seq | H3K9me3 | 100 bp PE | *D. melanogaster* | brain | Fig. 2-3, 5 | SRR12192012 |
| Female | 9 day | 3 | ChIP-Seq | H3K9me3 | 100 bp PE | *D. melanogaster* | brain | Fig. 2-3, 5 | SRR12192045 |
| Female | 9 day | 4 | ChIP-Seq | H3K9me3 | 100 bp PE | *D. melanogaster* | brain | Fig. 2-3, 5 | SRR12192034 |
| Female | 98 day | 1 | ChIP-Seq | H3K9me3 | 100 bp PE | *D. melanogaster* | brain | Fig. 2-3, 5 | SRR12192015 |
| Female | 98 day | 2 | ChIP-Seq | H3K9me3 | 100 bp PE | *D. melanogaster* | brain | Fig. 2-3, 5 | SRR12192014 |
| Female | 80 day | 3 | ChIP-Seq | H3K9me3 | 100 bp PE | *D. melanogaster* | brain | Fig. 2-3, 5 | SRR12192011 |
| Female | 80 day | 4 | ChIP-Seq | H3K9me3 | 100 bp PE | *D. melanogaster* | brain | Fig. 2-3, 5 | SRR12192010 |
| Female | 5 day | 1 | ChIP-Seq | Input | 100 bp PE | *D. melanogaster* | brain | Fig. 2-3, 5 | SRR12192023 |
| Female | 5 day | 2 | ChIP-Seq | Input | 100 bp PE | *D. melanogaster* | brain | Fig. 2-3, 5 | SRR12192018 |
| Female | 9 day | 3 | ChIP-Seq | Input | 100 bp PE | *D. melanogaster* | brain | Fig. 2-3, 5 | SRR12192017 |
| Female | 9 day | 4 | ChIP-Seq | Input | 100 bp PE | *D. melanogaster* | brain | Fig. 2-3, 5 | SRR12192016 |
| Female | 98 day | 1 | ChIP-Seq | Input | 100 bp PE | *D. melanogaster* | brain | Fig. 2-3, 5 | SRR12192053 |
| Female | 98 day | 2 | ChIP-Seq | Input | 100 bp PE | *D. melanogaster* | brain | Fig. 2-3, 5 | SRR12192052 |
| Female | 80 day | 3 | ChIP-Seq | Input | 100 bp PE | *D. melanogaster* | brain | Fig. 2-3, 5 | SRR12192051 |
| Female | 80 day | 4 | ChIP-Seq | Input | 100 bp PE | *D. melanogaster* | brain | Fig. 2-3, 5 | SRR12192050 |
| Male | 5 day | 1 | ChIP-Seq | H3K9me3 | 100 bp PE | *D. melanogaster* | brain | Fig. 2-3, 5 | SRR12192049 |
| Male | 5 day | 2 | ChIP-Seq | H3K9me3 | 100 bp PE | *D. melanogaster* | brain | Fig. 2-3, 5 | SRR12192048 |
| Male | 9 day | 3 | ChIP-Seq | H3K9me3 | 100 bp PE | *D. melanogaster* | brain | Fig. 2-3, 5 | SRR12192047 |
| Male | 9 day | 4 | ChIP-Seq | H3K9me3 | 100 bp PE | *D. melanogaster* | brain | Fig. 2-3, 5 | SRR12192046 |
| Male | 98 day | 1 | ChIP-Seq | H3K9me3 | 100 bp PE | *D. melanogaster* | brain | Fig. 2-3, 5 | SRR12192040 |
| Male | 98 day | 2 | ChIP-Seq | H3K9me3 | 100 bp PE | *D. melanogaster* | brain | Fig. 2-3, 5 | SRR12192039 |
| Male | 80 day | 3 | ChIP-Seq | H3K9me3 | 100 bp PE | *D. melanogaster* | brain | Fig. 2-3, 5 | SRR12192038 |
| Male | 82 day | 4 | ChIP-Seq | H3K9me3 | 100 bp PE | *D. melanogaster* | brain | Fig. 2-3, 5 | SRR12192037 |
| Male | 5 day | 1 | ChIP-Seq | Input | 100 bp PE | *D. melanogaster* | brain | Fig. 2-3, 5 | SRR12192044 |
| Male | 5 day | 2 | ChIP-Seq | Input | 100 bp PE | *D. melanogaster* | brain | Fig. 2-3, 5 | SRR12192043 |
| Male | 9 day | 3 | ChIP-Seq | Input | 100 bp PE | *D. melanogaster* | brain | Fig. 2-3, 5 | SRR12192042 |
| Male | 9 day | 4 | ChIP-Seq | Input | 100 bp PE | *D. melanogaster* | brain | Fig. 2-3, 5 | SRR12192041 |
| Male | 98 day | 1 | ChIP-Seq | Input | 100 bp PE | *D. melanogaster* | brain | Fig. 2-3, 5 | SRR12192036 |
| Male | 98 day | 2 | ChIP-Seq | Input | 100 bp PE | *D. melanogaster* | brain | Fig. 2-3, 5 | SRR12192035 |
| Male | 80 day | 3 | ChIP-Seq | Input | 100 bp PE | *D. melanogaster* | brain | Fig. 2-3, 5 | SRR12192033 |
| Male | 82 day | 4 | ChIP-Seq | Input | 100 bp PE | *D. melanogaster* | brain | Fig. 2-3, 5 | SRR12192032 |
