# Supplementary material for: Toxic Y chromosome: Increased repeat expression and age-associated heterochromatin loss in male Drosophila with a young Y chromosome
Source: PLoS Genet. 2021 Apr 22;17(4):e1009438. doi: 10.1371/journal.pgen.1009438 (PMC8061872; doi:10.1371/journal.pgen.1009438)
Supplement: S2 Table — Description of mapping statistics for ChIP-experiment data for each batch. Reported numbers of reads mapped to the sample and spike-in genomes. (PDF) [file pgen.1009438.s021.pdf]

**Table S2 - Mapping statistics of ChIP data**

Description of mapping statistics for ChIP-experiment data for each batch. Reported numbers of reads mapped to the sample and spike-in genomes.

| Sample | Total reads *post-trimming* | Reads map to *D. miranda* | Reads map to *D. melanogaster* | Total INPUT reads | Input reads map *D. miranda* | Input reads map *D. melanogaster* |
|---|---|---|---|---|---|---|
| Young Female 1 | 67,704,582 | 56,928,414 | 10,662,194 | 44,807,108 | 35,615,280 | 9,159,754 |
| Young Female 2 | 61,703,392 | 51,974,874 | 10,001,605 | 45,237,472 | 34,809,823 | 10,576,212 |
| Young Female 3 | 48,851,678 | 39,751,715 | 8,736,930 | 42,824,410 | 28,970,764 | 12,752,833 |
| Young Female 4 | 51,493,160 | 44,725,176 | 6,770,327 | 44,628,860 | 35,458,976 | 9,229,236 |
| Old Female 1 | 64,473,240 | 44,239,808 | 20,178,734 | 38,856,540 | 26,691,510 | 12,071,580 |
| Old Female 2 | 56,463,368 | 33,046,791 | 23,559,347 | 46,816,274 | 22,722,284 | 24,068,004 |
| Old Female 3 | 47,613,398 | 39,574,264 | 7,780,996 | 39,235,422 | 30,364,995 | 8,806,333 |
| Old Female 4 | 58,142,168 | 50,838,062 | 7,422,156 | 49,946,734 | 42,060,051 | 7,913,083 |
| Young Male 1 | 57,794,342 | 50,934,715 | 6,381,307 | 47,569,564 | 39,831,886 | 7,611,881 |
| Young Male 2 | 75,280,770 | 67,181,375 | 8,044,335 | 51,955,562 | 43,878,867 | 8,176,091 |
| Young Male 3 | 61,947,110 | 54,787,100 | 6,446,831 | 54,470,572 | 47,203,948 | 6,690,023 |
| Young Male 4 | 54,218,228 | 48,982,633 | 4,725,311 | 47,507,242 | 40,741,207 | 6,741,949 |
| Old Male 1 | 60,294,580 | 39,653,383 | 20,288,116 | 50,722,396 | 28,781,010 | 21,744,490 |
| Old Male 2 | 53,493,186 | 41,908,320 | 11,603,888 | 45,531,982 | 34,772,933 | 10,843,562 |
| Old Male 3 | 63,904,722 | 54,639,077 | 8,361,739 | 39,046,054 | 30,543,313 | 8,409,519 |
| Old Male 4 | 83,095,444 | 73,518,270 | 8,853,517 | 101,375,398 | 83,614,989 | 17,641,675 |
