# Supplementary material for: Toxic Y chromosome: Increased repeat expression and age-associated heterochromatin loss in male Drosophila with a young Y chromosome
Source: PLoS Genet. 2021 Apr 22;17(4):e1009438. doi: 10.1371/journal.pgen.1009438 (PMC8061872; doi:10.1371/journal.pgen.1009438)
Supplement: S3 Table — We used H3K9me3 enrichment in 5kb windows from the autosomes and X chromosomes to conduct Pearson correlations between each pair of replicates. Pearson correlations were calculated using the base R function cor.test(replicateA, replicateB, method =“pearson”). (PDF) [file pgen.1009438.s022.pdf]

Table S3. Pearson coefficients between replicates of same biological sample

We used H3K9me3 enrichment in 5kb windows from the autosomes and X chromosomes to conduct Pearson correlations between each pair of replicates. Pearson correlations were calculated using the base R function cor.test(replicateA, replicateB, method=”pearson”).

		Replicate 1	Replicate 2	Replicate 3	Replicate 4
Young female	Replicate 1		0.9806	0.9709	0.9716
	Replicate 2	0.9806		0.9523	0.9554
	Replicate 3	0.9709	0.9523		0.9897
	Replicate 4	0.9716	0.9554	0.9897	
Old female	Replicate 1		0.9303	0.9654	0.9799
	Replicate 2	0.9303		0.9209	0.9313
	Replicate 3	0.9654	0.9209		0.9831
	Replicate 4	0.9799	0.9313	0.9831	
Young male	Replicate 1		0.9838	0.9614	0.9735
	Replicate 2	0.9838		0.9601	0.9711
	Replicate 3	0.9614	0.9601		0.9748
	Replicate 4	0.9735	0.9711	0.9748	
Old male	Replicate 1		0.8927	0.8890	0.8927
	Replicate 2	0.8927		0.9280	0.9347
	Replicate 3	0.8890	0.9280		0.9830
	Replicate 4	0.8927	0.9347	0.9830	
