# Supplementary material for: Toxic Y chromosome: Increased repeat expression and age-associated heterochromatin loss in male Drosophila with a young Y chromosome
Source: PLoS Genet. 2021 Apr 22;17(4):e1009438. doi: 10.1371/journal.pgen.1009438 (PMC8061872; doi:10.1371/journal.pgen.1009438)
Supplement: S4 Table — For each normalization method, we identified the top 20% most enriched 5kb windows in euchromatin (N = 4646). We then identified how many 5kb regions were shared between each normalization method and reported those results as a percentage (no. windows shared between two methods / no. windows in top 20% enrichment). (PDF) [file pgen.1009438.s023.pdf]

Table S4. Proportion of 5kb-windows in top 20 percentile H3K9me3 enrichment shared between normalization methods

For each normalization method, we identified the top 20% most enriched 5kb windows in euchromatin (N = 4646). We then identified how many 5kb regions were shared between each normalization method and reported those results as a percentage (no. windows shared between two methods / no. windows in top 20% enrichment).

Normalization method	No Spike-In	Brown (2020)	Bonhoure (2014)	Wei (2020)
No Spike-In		99.98%	99.53%	99.14%
Brown (2020)	99.98%		99.53%	99.16%
Bonhoure (2014)	99.53%	99.53%		99.61%
Wei (2020)	99.14%	99.16%	99.61%	
