# Supplementary material for: Toxic Y chromosome: Increased repeat expression and age-associated heterochromatin loss in male Drosophila with a young Y chromosome
Source: PLoS Genet. 2021 Apr 22;17(4):e1009438. doi: 10.1371/journal.pgen.1009438 (PMC8061872; doi:10.1371/journal.pgen.1009438)
Supplement: S5 Table — (PDF) [file pgen.1009438.s024.pdf]

Table S5. Mapping statistics of ChIP data for MAPQ > 3 alignments

Sample	Total reads *post-trimming*	Reads map to *D. miranda*	Reads map to *D. melanogaster*	Total INPUT reads *post-trimming*	Input reads map *D. miranda*	Input reads map *D. melanogaster*
Young Female 1	67,704,582	40,487,182	6,632,869	44,807,108	29,438,452	7,071,855
Young Female 2	61,703,392	36,914,280	6,312,139	45,237,472	28,552,858	8,250,055
Young Female 3	48,851,678	28,773,644	5,897,662	42,824,410	24,090,157	9,866,222
Young Female 4	51,493,160	31,849,850	4,396,533	44,628,860	29,431,898	6,973,192
Old Female 1	64,473,240	31,467,019	13,417,446	38,856,540	22,071,447	9,532,316
Old Female 2	56,463,368	24,217,457	15,790,171	46,816,274	22,071,447	19,356,315
Old Female 3	47,613,398	28,996,177	5,153,463	39,235,422	25,370,450	6,730,047
Old Female 4	58,142,168	36,984,324	4,719,688	49,946,734	35,064,663	5,898,510
Young Male 1	57,794,342	32,145,574	3,976,855	47,569,564	28,468,786	5,923,154
Young Male 2	75,280,770	42,501,933	5,085,807	51,955,562	31,322,018	6,391,210
Young Male 3	61,947,110	35,698,519	4,251,804	54,470,572	35,083,391	5,080,061
Young Male 4	54,218,228	31,554,397	3,042,336	47,507,242	29,534,573	5,160,270
Old Male 1	60,294,580	27,642,556	13,548,267	50,722,396	20,775,505	17,466,283
Old Male 2	53,493,186	27,186,663	7,733,141	45,531,982	24,972,759	8,654,831
Old Male 3	63,904,722	36,296,614	5,494,746	39,046,054	22,270,233	6,465,585
Old Male 4	83,095,444	48,664,557	5,924,032	101,375,398	60,903,326	13,563,320
