# Supplementary material for: Toxic Y chromosome: Increased repeat expression and age-associated heterochromatin loss in male Drosophila with a young Y chromosome
Source: PLoS Genet. 2021 Apr 22;17(4):e1009438. doi: 10.1371/journal.pgen.1009438 (PMC8061872; doi:10.1371/journal.pgen.1009438)
Supplement: S6 Table — (PDF) [file pgen.1009438.s025.pdf]

**Table S6. Heterochromatin-related genes and their expression change in old males and female**

| | | | Old Males | | | Old Females | | |
|---|---|---|---|---|---|---|---|---|
| FlybaseID | Gene Name | GO Biological Process | Regulation | Log2(fold-change) | Adj. P-val | Regulation | Log2(fold-change) | Adj. P-val |
| FBgn0246261 | Atf-2 | nuclear heterochromatin | up | 0.540 | **0.014** | up | 0.098 | 0.821 |
| FBgn0074791 | Trf2 | nuclear heterochromatin | down | 0.250 | 0.154 | up | 0.007 | 0.989 |
| FBgn0074758 | MED26 | nuclear heterochromatin | up | 0.102 | 0.904 | up | 0.614 | 0.721 |
| FBgn0080711 | MED17 | nuclear heterochromatin | up | 0.195 | 0.375 | up | 0.080 | 0.840 |
| FBgn0249854 | HP4 | nuclear heterochromatin | down | 0.084 | 0.859 | up | 0.130 | 0.818 |
| FBgn0247702 | moon | nuclear heterochromatin | up | 0.602 | 0.383 | up | 0.897 | *NA* |
| FBgn0244273 | Hmt4-20 | nuclear heterochromatin | down | 0.235 | 0.101 | down | 0.118 | 0.775 |
| FBgn0261712 | SuUR | nuclear heterochromatin | up | 0.119 | 0.609 | up | 0.169 | 0.749 |
| FBgn0078743 | Sirt1 | nuclear heterochromatin | down | 0.169 | 0.348 | up | 0.022 | 0.946 |
| FBgn0078701 | TfIIA-S | nuclear heterochromatin | up | 0.344 | 0.113 | up | 0.514 | 0.114 |
| FBgn0081044 | HP1A | nuclear heterochromatin | up | 0.199 | 0.499 | up | 0.091 | 0.842 |
| FBgn0071193 | Arp6 | heterochromatin organization | down | 0.584 | 0.003 | down | 0.436 | 0.087 |
| FBgn0078014 | vig | heterochromatin organization | down | 0.375 | 0.107 | down | 0.121 | 0.848 |
| FBgn0071285 | vig2 | heterochromatin organization | down | 0.018 | 0.960 | up | 0.175 | 0.699 |
| FBgn0265021 | egg | heterochromatin organization | up | 0.124 | 0.573 | down | 0.036 | 0.939 |
| FBgn0074378 | Su(var)3-3 | heterochromatin organization | up | 0.102 | 0.652 | down | 0.072 | 0.847 |
| FBgn0074067 | hop | heterochromatin assembly | up | 0.109 | 0.641 | up | 0.037 | 0.931 |
| FBgn0079367 | piwi | heterochromatin assembly | down | 0.658 | 0.463 | up | 0.320 | 0.739 |
| FBgn0247875 | Stat92E | heterochromatin assembly | up | 0.213 | 0.098 | up | 0.356 | 0.113 |
| FBgn0080534 | Mekk1 | heterochromatin assembly | up | 0.160 | 0.380 | up | 0.026 | 0.943 |
| FBgn0078103 | Bap60 | heterochromatin assembly | up | 0.103 | 0.723 | up | 0.028 | 0.943 |
| FBgn0246408 | wde | heterochromatin assembly | up | 0.247 | 0.187 | down | 0.085 | 0.802 |
| FBgn0075184 | Acf | heterochromatin assembly | up | 0.008 | 0.978 | down | 0.114 | 0.682 |
| FBgn0248173 | htt | heterochromatin assembly | down | 0.058 | 0.818 | down | 0.166 | 0.516 |
| FBgn0078708 | Fmr1 | heterochromatin assembly | up | 0.237 | 0.085 | up | 0.186 | 0.521 |
| FBgn0080049 | HP1b | heterochromatin assembly | down | 0.139 | 0.563 | down | 0.117 | 0.804 |
| FBgn0245751 | HPS4 | heterochromatin assembly | up | 0.402 | 0.088 | up | 0.293 | 0.331 |
| FBgn0080124 | CG7137 | heterochromatin assembly | up | 0.138 | 0.705 | down | 0.097 | 0.822 |
| FBgn0081790 | FBXO11 | heterochromatin assembly | down | 0.050 | 0.852 | down | 0.053 | 0.904 |
| FBgn0079435 | CG6204 | heterochromatin assembly | down | 0.162 | 0.440 | down | 0.109 | 0.758 |
| FBgn0080459 | Naa40 | heterochromatin assembly | down | 0.048 | 0.893 | up | 0.167 | 0.683 |
| FBgn0077229 | Charc-14 | heterochromatin assembly | up | 0.240 | 0.418 | up | 0.010 | 0.985 |
| FBgn0244102 | CoRest | heterochromatin assembly | up | 0.140 | 0.495 | up | 0.034 | 0.917 |
| FBgn0070268 | gpp | heterochromatin assembly | down | 0.114 | 0.518 | up | 0.171 | 0.756 |
