# Supplementary material for: Toxic Y chromosome: Increased repeat expression and age-associated heterochromatin loss in male Drosophila with a young Y chromosome
Source: PLoS Genet. 2021 Apr 22;17(4):e1009438. doi: 10.1371/journal.pgen.1009438 (PMC8061872; doi:10.1371/journal.pgen.1009438)
Supplement: S7 Table — Description of samples/tissues used for 3 rounds/batches of RNA---seq experiments. (PDF) [file pgen.1009438.s026.pdf]

Table S7 --- Overview of RNA data generated

Description of samples/tissues used for 3 rounds/batches of RNA---seq experiments.

Sex	Age	Batch	Data type	Tissue	reads	Figure	reads generated	SRA Accession
Female	9 day	1	RNA-Seq	pooled brains	100 bp PE	Fig. 5-6	45,436,492	SRR12192031
Female	9 day	2	RNA-Seq	pooled brains	100 bp PE	Fig. 5-6	149,817,434	SRR12192030
Female	9 day	3	RNA-Seq	pooled brains	100 bp PE	Fig. 5-6	179,126,850	SRR12192029
Female	80 day	1	RNA-Seq	pooled brains	100 bp PE	Fig. 5-6	60,918,286	SRR12192028
Female	80 day	2	RNA-Seq	pooled brains	100 bp PE	Fig. 5-6	150,266,062	SRR12192027
Female	80 day	3	RNA-Seq	pooled brains	100 bp PE	Fig. 5-6	168,272,836	SRR12192026
Male	9 day	1	RNA-Seq	pooled brains	100 bp PE	Fig. 5-6	54,347,756	SRR12192025
Male	9 day	2	RNA-Seq	pooled brains	100 bp PE	Fig. 5-6	136,139,696	SRR12192024
Male	9 day	3	RNA-Seq	pooled brains	100 bp PE	Fig. 5-6	144,374,744	SRR12192022
Male	80 day	1	RNA-Seq	pooled brains	100 bp PE	Fig. 5-6	45,924,434	SRR12192021
Male	80 day	2	RNA-Seq	pooled brains	100 bp PE	Fig. 5-6	152,547,756	SRR12192020
Male	80 day	3	RNA-Seq	pooled brains	100 bp PE	Fig. 5-6	159,981,252	SRR12192019
