# Supplementary material for: Toxic Y chromosome: Increased repeat expression and age-associated heterochromatin loss in male Drosophila with a young Y chromosome
Source: PLoS Genet. 2021 Apr 22;17(4):e1009438. doi: 10.1371/journal.pgen.1009438 (PMC8061872; doi:10.1371/journal.pgen.1009438)
Supplement: S8 Table — Description of mapping statistics for RNA-seq experiment data for each batch. Reported gene and repeat counts by Subread featurecounts and TEtranscripts where applicable. (PDF) [file pgen.1009438.s027.pdf]

Table S8 - Mapping and counts statistics of RNA data

Description of mapping statistics for RNA-seq experiment data for each batch. Reported gene and repeat counts by Subread featurecounts and TEtranscripts where applicable.

Sample	Total reads *post-rRNA removal*	Reads map to *D. miranda*	Gene count totals by		Transposable element count totals by				Satellite DNA count totals by	
			Subread featurecounts	*TEtranscripts*	*Subread featurecounts*	%	*TEtranscripts*	%	*Subread featurecounts*	%
YF1	33,396,824	30,496,555	14,091,845	7,225,056	473,202	1.6%	400,725	1.3%	28,386	0.09%
YF2	127,413,478	119,301,094	56,069,961	28,333,230	2,523,245	2.1%	1,786,219	1.5%	137,691	0.12%
YF3	161,923,358	151,229,688	70,388,358	35,588,522	2,689,933	1.8%	2,018,726	1.3%	172,596	0.11%
OF1	21,683,064	18,353,375	8,635,458	4,454,130	236,941	1.3%	201,467	1.1%	15,466	0.08%
OF2	132,515,030	124,508,964	59,588,781	29,818,464	2,103,645	1.7%	1,531,115	1.2%	143,510	0.12%
OF3	121,338,760	99,588,981	47,480,007	23,937,653	1,223,690	1.2%	967,402	1.0%	98,327	0.10%
YM1	33,184,830	30,019,808	13,223,662	6,768,926	1,070,610	3.6%	597,786	2.0%	37,394	0.12%
YM2	125,532,740	115,020,312	51,002,631	24,791,212	5,271,178	4.6%	2,611,345	2.3%	198,342	0.17%
YM3	132,522,320	120,583,108	55,586,184	27,626,956	4,356,357	3.6%	2,234,565	1.9%	186,127	0.15%
OM1	25,670,500	22,481,303	10,001,558	6,768,926	871,614	3.9%	468,626	2.1%	28,016	0.12%
OM2	132,662,164	123,016,943	55,405,264	27,935,265	5,066,965	4.1%	2,488,609	2.0%	177,217	0.14%
OM3	141,307,748	131,079,565	61,138,923	30,732,062	4,270,558	3.3%	2,149,597	1.6%	178,617	0.14%
