# Supplementary material for: Toxic Y chromosome: Increased repeat expression and age-associated heterochromatin loss in male Drosophila with a young Y chromosome
Source: PLoS Genet. 2021 Apr 22;17(4):e1009438. doi: 10.1371/journal.pgen.1009438 (PMC8061872; doi:10.1371/journal.pgen.1009438)
Supplement: S9 Table — (PDF) [file pgen.1009438.s028.pdf]

Table S9. Mapping and counts statistics of RNA data filtered for MAPQ > 40

Sample	Reads map to	Gene count totals by		Transposable element count totals by				Satellite DNA count totals by
	D. miranda	*Subread featurecounts*	*TEtranscripts*	*Subread featurecounts*	*%*	*TEtranscripts*	*%*	*Subread featurecounts*
YF1	24,470,598	14,091,845	7,069,630	473,368	1.9%	297,510	1.2%	28,386
YF2	95,902,373	56,069,961	27,745,752	2,523,245	2.6%	1,303,256	1.4%	137,691
YF3	123,024,250	70,388,358	34,892,990	2,689,933	2.2%	1,542,943	1.3%	172,596
OF1	14,148,032	8,635,458	4,361,564	237,119	1.7%	147,776	1.0%	15,466
OF2	97,593,658	59,588,781	29,207,255	2,103,645	2.2%	1,146,703	1.2%	143,510
OF3	76,595,230	47,480,007	23,467,539	1,223,690	1.6%	741,431	1.0%	98,327
YM1	23,401,050	13,223,662	6,583,993	1,070,996	4.6%	403,013	1.7%	37,394
YM2	92,043,043	51,002,631	24,071,978	5,271,178	5.7%	1,790,176	1.9%	198,342
YM3	96,286,901	55,586,184	26,862,946	4,356,357	4.5%	1,548,567	1.6%	186,127
OM1	16,957,126	10,001,558	4,980,605	872,043	5.1%	317,498	1.9%	28,016
OM2	93,999,119	55,405,264	27,124,819	5,066,965	5.4%	1,670,406	1.8%	177,217
OM3	99,173,506	61,138,923	29,883,477	4,270,558	4.3%	1,468,000	1.5%	178,617
