# Supplementary material for: Toxic Y chromosome: Increased repeat expression and age-associated heterochromatin loss in male Drosophila with a young Y chromosome
Source: PLoS Genet. 2021 Apr 22;17(4):e1009438. doi: 10.1371/journal.pgen.1009438 (PMC8061872; doi:10.1371/journal.pgen.1009438)
Supplement: S10 Table — (PDF) [file pgen.1009438.s029.pdf]

Table S10 DNAPipeTE estimates of repeats in RNASeq data

	% TE	Average	% All repeats	Average
Young Female 2	28.1%	21.5%	46.0%	38.3%
Young Female 3	19.9%		36.1%	
Young Female 1	16.6%		32.80%	
Old Female 2	20.8%	22.4%	37.60%	36.5%
Old Female 3	29.9%		41.50%	
Old Female 1	16.5%		30.40%	
Young Male 2	33.4%	23.7%	49.70%	40.3%
Young Male 3	18.9%		35.60%	
Young Male 1	18.7%		35.50%	
Old Male 2	19.3%	23.2%	38.10%	39.2%
Old Male 3	30.7%		44.60%	
Old Male 1	19.5%		34.80%	
