# Supplementary material for: Toxic Y chromosome: Increased repeat expression and age-associated heterochromatin loss in male Drosophila with a young Y chromosome
Source: PLoS Genet. 2021 Apr 22;17(4):e1009438. doi: 10.1371/journal.pgen.1009438 (PMC8061872; doi:10.1371/journal.pgen.1009438)
Supplement: S1 Appendix — (PDF) [file pgen.1009438.s030.pdf]

S1 Appendix. *D. melanogaster* as a spike-in to normalize *D. miranda* ChIP's

The use of a spike-in allows normalization of individual ChIP experiments, and enables us to compare levels of H3K9me3 enrichment across sexes and time. We chose *D. melanogaster* to serve as a spike-in for *D. miranda*, and below, we demonstrate that this species pair is sufficiently diverged from each other that there is very little ambiguity in the assignment of reads to the correct species.

Table A1 shows how many of the reads that were originally mapped to *D. miranda* also mapped to *D. melanogaster*. Specifically, we took reads that initially mapped to *D.miranda* and mapped them to the *D. miranda* and the *D.melanogaster* genomes simultaneously to estimate the cross-mapping rate (same Bowtie2 parameters as initial mappings). We find that, on average, only about 0.6% of the reads that initially aligned to *D. miranda* will cross-map to *D. melanogaster.*

Table A1. Cross-mapping statistics of *D. miranda* ChIP-Seq reads to *D. melanogaster*

Sample	Reads map to *D. miranda*	Cross-mapping *to D. melanogaster*	Effective Rate	Input reads map *D. miranda*	Cross-mapping *to D. melanogaster*	Effective Rate
YF1	56,928,414	412,013	0.7%	35,615,280	276,386	0.8%
YF2	51,974,874	391,147	0.8%	34,809,823	280,416	0.8%
YF3	39,751,715	284,123	0.7%	28,970,764	249,567	0.9%
YF4	44,725,176	309,167	0.7%	35,458,976	271,407	0.8%
OF1	44,239,808	539,401	1.2%	26,691,510	264,336	1.0%
OF2	33,046,791	533,892	1.6%	22,722,284	355,757	1.6%
OF3	39,574,264	301,770	0.8%	30,364,995	233,662	0.8%
OF4	50,838,062	381,440	0.8%	42,060,051	292,726	0.7%
YM1	50,934,715	128,219	0.3%	39,831,886	114,441	0.3%
YM2	67,181,375	164,696	0.2%	43,878,867	123,689	0.3%
YM3	54,787,100	116,963	0.2%	47,203,948	99,499	0.2%
YM4	48,982,633	101,850	0.2%	40,741,207	99,723	0.2%
OM1	39,653,383	294,845	0.7%	28,781,010	230,548	0.8%
OM2	41,908,320	187,926	0.4%	34,772,933	138,877	0.4%
OM3	54,639,077	135,974	0.2%	30,543,313	100,235	0.3%
OM4	73,518,270	160,215	0.2%	83,614,989	228,989	0.3%

We similarly mapped genomic DNA from *D. miranda* to only the *D. melanogaster* genome using the same parameters as described previously. We found that of 24,375,599 male reads mapped to D. miranda, 275,936 (0.1%) then mapped to D. melanogaster and of the 15,563,664 female reads mapped to D. miranda, 151,165(1%) mapped to D. melanogaster. These cross-mapping

estimates are similar to those in the ChIP-data, suggesting that any reads mapped to *D. miranda* are likely derived from *D. miranda*.

Table A2 shows cross-mapping of reads derived from *D. miranda* heterochromatin. We took reads originally mapped to *D. miranda* heterochromatin and mapped them simultaneously to both *D. miranda* and *D. melanogaster* genomes to infer cross-mapping between the species' repeats (same Bowtie2 parameters). We find that the rate is almost always less than 1% (especially when omitting Old Male Replicate 1) and this suggests low cross-mapping between species-specific repeats. Lower cross-mapping from heterochromatic reads between *D. miranda* and *D. melanogaster* presumably reflects the rapid turn-over of repetitive DNA between species (that is, repeats are typically less conserved between species than unique DNA).

Table A2. Cross-mapping statistics of *D. miranda* ChIP-Seq reads from heterochromatin

Sample	Sample reads	Reads re-map to *D. miranda*	Cross-mapping *to D. melanogaster*	Effective Rate	Input reads	Input reads re-map to *D. miranda*	Cross-mapping *to D. melanogaster*	Effective Rate
YF1	20,311,258	20,301,290	8,617	0.0%	6,318,194	6,313,904	3,935	0.1%
YF2	17,182,960	17,173,800	8,223	0.0%	5,871,858	5,866,876	4,527	0.1%
YF3	14,143,700	14,138,560	2,903	0.0%	5,164,536	5,159,248	4,771	0.1%
YF4	16,697,004	16,692,800	2,515	0.0%	6,433,086	6,429,049	3,617	0.1%
OF1	15,233,926	15,216,770	16,238	0.1%	4,893,508	4,888,142	5,115	0.1%
OF2	9,833,772	9,814,370	18,684	0.2%	3,881,778	3,871,189	10,218	0.3%
OF3	12,951,250	12,946,530	2,667	0.0%	5,381,206	5,377,031	3,686	0.1%
OF4	16,713,484	16,708,360	3,492	0.0%	7,391,458	7,387,815	3,168	0.0%
YM1	28,196,674	28,084,860	106,150	0.4%	17,060,460	16,971,160	87,009	0.5%
YM2	36,895,602	36,754,400	135,483	0.4%	18,646,480	18,550,720	93,402	0.5%
YM3	29,207,016	29,096,950	99,585	0.3%	18,285,618	18,201,040	78,574	0.4%
YM4	26,575,570	26,479,620	87,559	0.3%	17,185,284	17,102,750	79,363	0.5%
OM1	18,448,058	18,219,970	224,821	1.2%	11,966,652	11,811,810	153,172	1.3%
OM2	21,710,342	21,561,380	145,969	0.7%	14,545,924	14,445,320	98,874	0.7%
OM3	27,623,332	27,498,560	113,585	0.4%	12,708,820	12,629,980	76,299	0.6%
OM4	36,853,832	36,710,190	133,561	0.4%	34,771,568	34,590,330	176,644	0.5%
