# Supplementary material for: Toxic Y chromosome: Increased repeat expression and age-associated heterochromatin loss in male Drosophila with a young Y chromosome
Source: PLoS Genet. 2021 Apr 22;17(4):e1009438. doi: 10.1371/journal.pgen.1009438 (PMC8061872; doi:10.1371/journal.pgen.1009438)
Supplement: S2 Appendix — (PDF) [file pgen.1009438.s031.pdf]

S2 Appendix. Cross-mapping between the *D. miranda* neo-X and neo-Y chromosome

The neo-Y is recently diverged from the neo-X and thus may potentially produce mis-mapping issues. Several lines of evidence suggest that mis-mapping between the neo-sex chromosomes is of limited concern. **Table B1** shows how many reads that originally mapped to the neo-Y re-map to either the neo-X or the neo-Y by mapping to both contigs simultaneously with Bowtie2. We find that roughly 96% of reads originally mapped to the neo-Y will map back to the neo-Y while only 1% will map to the neo-X. These data suggest that cross-mapping between the two chromosomes is very low since the vast majority of reads will align back to the original chromosome.

Table B1. Cross-mapping statistics of neo-Y vs neo-X reads

Sample	ChIP reads mapped *to neo-Y*	Mapped back *to neo-Y*	Cross-mapped *to neo-X*	Cross-mapping rate	Input reads mapped *neo-Y*	Mapped back *to neo-Y*	Cross-mapped *to neo-X*	Cross-mapping rate
YM1	19,693,004	19,005,755	263,436	1.4%	14,449,500	14,101,122	164,634	1.2%
YM2	25,598,614	24,865,384	358,057	1.4%	15,687,486	15,304,489	188,379	1.2%
YM3	21,532,467	20,417,131	275,963	1.3%	15,756,929	15,008,153	173,940	1.1%
YM4	19,203,518	18,298,989	252,029	1.4%	14,585,246	14,177,026	161,838	1.1%
OM1	15,055,685	14,625,256	167,524	1.1%	10,219,496	9,964,189	112,765	1.1%
OM2	16,286,668	15,868,116	214,098	1.3%	12,230,167	11,940,836	147,798	1.2%
OM3	21,781,736	20,563,365	266,817	1.3%	10,803,142	10,481,635	120,293	1.1%
OM4	29,375,200	27,726,419	362,116	1.3%	29,685,148	28,798,995	331,412	1.1%

We also simulated 100-bp paired-end reads from the neo-X and the neo-Y using ART (Huang et al. 2012) and mapped both of these simulated read sets to the neo-X and the neo-Y. The following parameters were used: -ss HS20 -l 100 -f 10 -p -m 375 -s 25. **Table B2** shows that overall, the vast majority of reads maps back to their respective chromosome and even more so when we apply an alignment quality cutoff. In particular, over 99% of simulated neo-Y reads map back to the neo-Y (and 99.9% of reads if using a MAPQ>3).

Table B2. Cross-mapping statistics between neo-X and neo-Y using simulated reads.

	All mappings				Mappings above MAPQ > 3			
	Total reads	Map to neo-X	Map to neo-Y	Cross-mapping %	Total reads	Map to neo-X	Map to neo-Y	Cross-mapping %
Neo-X	2,530,344	2,443,493	86,851	3.432%	2,247,137	2,235,727	11,410	0.51%
Neo-Y	11,041,076	89,021	10,952,055	0.806%	7,535,923	10,685	7,525,238	0.14%

We further assessed mis-mapping from the neo-X to the neo-Y by mapping female genomic DNA reads from D. *miranda*. We mapped female gDNA reads [parameters] and mapped them to the female genome; we then took all reads mapping to the neo-X and re-mapped them to both the neo-X and the neo-Y. Of the original 2,042,127 neo-X alignments, 2,020,692 (98.9%) were aligned properly to the neo-X and the rest (1.1%) aligned to the neo-Y. Thus, similar to our results of mapping neo-Y reads to the neo-X, the vast majority of reads will be reassigned to their initial chromosome.
