# Supplementary material for: Toxic Y chromosome: Increased repeat expression and age-associated heterochromatin loss in male Drosophila with a young Y chromosome
Source: PLoS Genet. 2021 Apr 22;17(4):e1009438. doi: 10.1371/journal.pgen.1009438 (PMC8061872; doi:10.1371/journal.pgen.1009438)
Supplement: S17 Fig — Left: (Top) Satellites in young females v. young males, (bottom) satellites in old females vs old males. Fold change is indicative of male/female. Right: (Top) Satellites in young males vs old males, (bottom) satellites young females vs old females. Fold change is indicative of young/old. (PDF) [file pgen.1009438.s017.pdf]

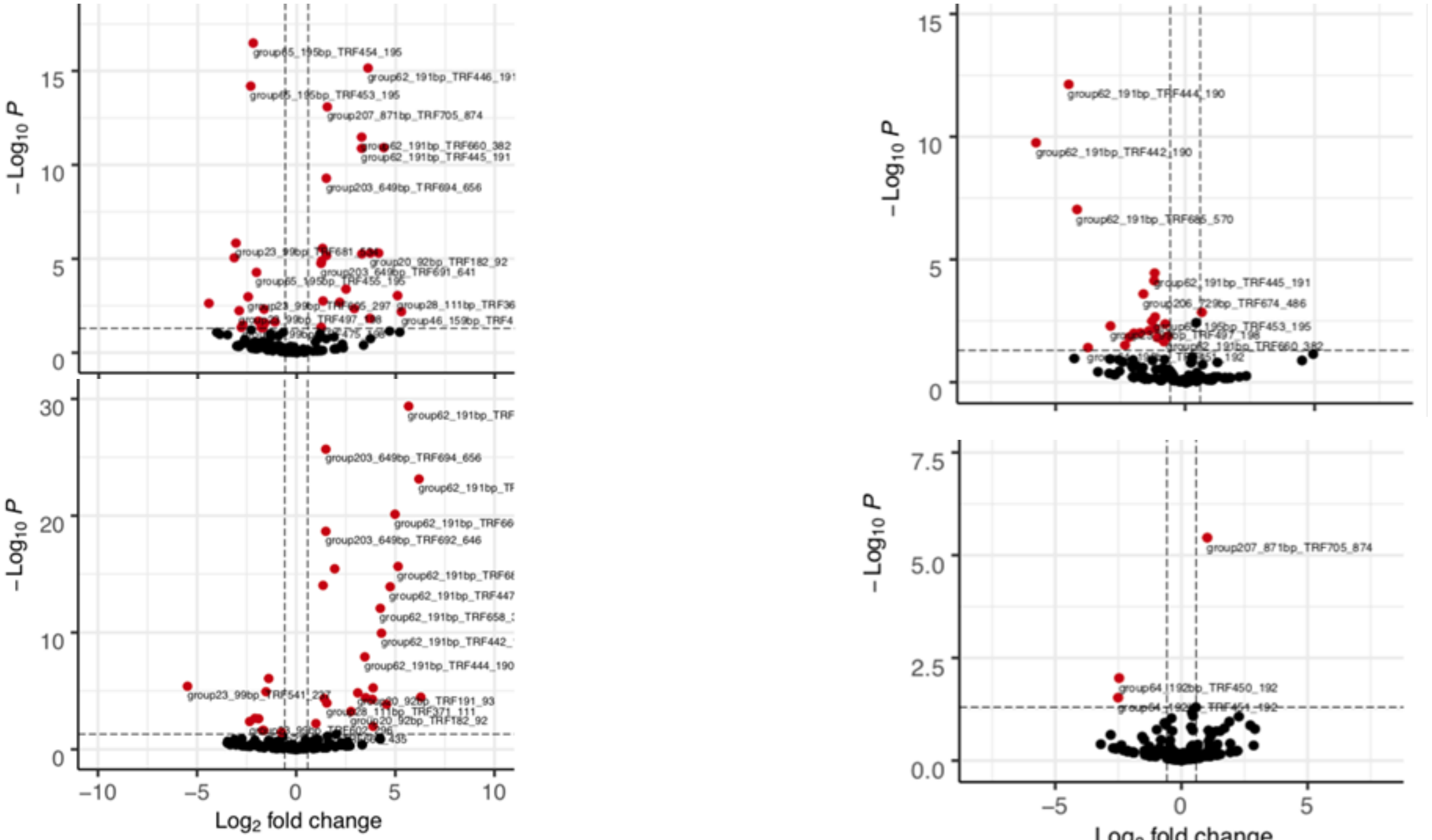

Figure S17: Volcano plots for satellite DNA expression plots by sex and age.
