# Supplementary material for: Toxic Y chromosome: Increased repeat expression and age-associated heterochromatin loss in male Drosophila with a young Y chromosome
Source: PLoS Genet. 2021 Apr 22;17(4):e1009438. doi: 10.1371/journal.pgen.1009438 (PMC8061872; doi:10.1371/journal.pgen.1009438)
Supplement: Attachment — Submitted filename: 0_CoverLetter.docx [file pgen.1009438.s032.docx]

Dear Dr. Hawley,

Please find enclosed a substantially revised and improved version of our manuscript 'Toxic Y chromosome: increased repeat expression and age-associated heterochromatin loss in male Drosophila with a young Y chromosome'. We have incorporated all of the suggestions of the reviewers, which made this a stronger paper. In particular, we performed orthogonal validation that H3K9me3 is indeed lower in the neo-Y chromosome (Figure 3B, Figure S10), and demonstrate that our analysis is robust with respect to repetitive sequences (Appendix 1 and 2, and several new Supplementary Figures). We enclose a detailed list of our responses to the reviewer’s comments below. We hope that you find this revised version suitable for publication in *PLoS Genetics*.

Yours sincerely,

Doris Bachtrog

**Response to Reviewer #1:**

In this study, Nguyen and Bachtrog addressed an outstanding question in evolutionary genomics – how does the evolution of sex chromosome influence epigenomes and, accordingly, individual phenotypes ("aging"). Studies from the Bachtrog lab previously reported that, compared to females, silenced transposable elements (TEs) and satellite repeats are derepressed in aged D. melanogaster males due to the "toxic Y" effect. In this study, authors reported that in a species with recently evolved neo-Y chromosome, D. miranda, there is similar derepression of TEs and satellite repeats in males. However, the derepression of TEs is not restricted to aged males, but also present in young males. Authors suggested that the much larger neo-Y chromosome is a bigger "burden" and thus more toxic. Another major finding from the study is that, compared to other heterochromatic regions, neo-Y has lower enrichment of heterochromatic marks, and the loss of heterochromatin differs between males and females. While these findings are exciting and could provide new insights for the field, there are several potential caveats that need to be formally addressed to ensure the robustness of the authors' findings. Below I listed my specific comments.

We thank the reviewer for his/her assessment and detailed comments, which make this a stronger paper.

1. Most of the major conclusions from this study were drawn from the analysis of H3K9me3 enrichment and the expression of repetitive sequences (satellite repeats or TEs). Because of their repetitive nature, reads from these sequences will appear as "multi-mapped reads" in ChIP-seq or RNA-seq data. However, the authors did not provide enough details about how they address the multiple-mapping issue.

Some of the reported analyses made me especially confused. For instance, in page 6, the authors stated that the results are consistent when using 5kb windows with different percentages of repetitive sequences, even for windows with >99.5% repetitive sequences. This makes it sound like that the authors only focused on unique sequences when estimating H3K9me3 enrichment (but also see below). However, 0.005*5,000 = 25, meaning that the H3K9me3 estimates were based on merely 25bp of unique sequence?

In order to increase mappability of our ChIP-seq data (both to repetitive regions, and to distinguish between the neo-X and neo-Y), we collected 100-bp paired-end read data. We used alignments to the entire genome, which were performed using Bowtie2. Using these alignments, we calculated H3K9me3 enrichment with sliding 5kb-windows across the genome. For the specific result in question, we categorized these windows by the amount of repetitive sequences (that is, % of bp that are repeat-masked in that window) to aggregate H3K9me3 enrichment by the repeat-content of the window. In this manner, the H3K9me3 estimates binned by % repeats are not based on 25bp of sequence but rather the alignment of 100bp paired-end reads to locations in the genome (which are later binned by repeat %). Importantly, even if a region is considered 100% repetitive (i.e. all the bp were repeat-masked), it still will contain unique SNP’s or a unique arrangement of repeats that will allow a sizeable fraction of reads to map uniquely to such a region. As detailed in our revision (see comments below), we make sure that our results are robust with regards to mapping to repetitive DNA.

If authors only used unique sequences, it is unclear what the filtering criteria are (e.g., mapping quality). If authors allowed multi-mapped reads, then how did bowtie assign multi-mapped reads, or how authors decided where a repetitive read came from?

In the main figures, we use the alignments reported from *Bowtie2* and in Figure S3 we use reads above a quality cutoff (MAPQ > 3) to only consider uniquely mapping reads. According to the *Bowtie2* manual, *Bowtie2* will find the best locations for each read and for reads that map equally well to multiple locations it will randomly assign the read to one of these locations. Therefore reads that are multi-mapped are reported once and their abundance is ‘averaged’ across their genomic locations. In Figure S3, we kept alignments with a MAPQ score above 3 to filter out multi-mapped reads (which are assigned highest MAPQ of 1) and to retain reads with up to 3 mismatches.

For HISAT2 mappings, we used all reported alignments from the default option. We now provide TE and satellite counts from alignments whose MAPQ > 40 in **Table S9**, which again eliminates multiply mapping reads. Overall, we still observe more TE abundance in male RNASeq data compared to female data.

We also provide a non-library mapping based approach to estimate TE abundance in our RNASeq data by using *dnaPipeTE*. Briefly, this software subsamples the data to low coverage and infers if reads are single-copy (i.e. euchromatic regions) or multi-copy (i.e. repeats). We provide the results of this denovo-based estimation approach in **Table S10**. Again, we find that males generally have a higher proportion of their gene expression derived from TEs, and repeats in general, compared to females.

The authors also compared the H3K9me3 enrichment level between TEs on the neo-Y and other pericentromeric heterochromatin. How did the authors distinguish repetitive TEs in different genomic locations? Did they use the H3K9me3 enrichment in the unique flanking sequence (if so, data need to be shown)?

We distinguished H3K9me3 enrichment at repetitive TEs in different genomic locations by mapping all paired-end reads to the entire genome with *Bowtie2*. In this manner, all alignments considered are mapped with specificity to a particular genomic location. Then, we used *TEtranscripts*, a software that estimates TE counts by using whole-genome alignments and repeat-locations on a genome, to calculate H3K9me3 enrichment levels between TEs on the neo-Y and elsewhere.

In addition, how the RNA-seq comparison is normalized with respect to TE copy number is not clear. Especially, there are TE copies inside and outside heterochromatin regions. How the authors distinguished repetitive copies within and outside heterochromatin (and even more, on neo-Y and other chromosomes)?

We estimated TE copy number by accounting for genome-wide copies, irrespective of being in heterochromatin or euchromatin, and irrespective of the genome assembly, by mapping genomic DNA reads to the reference TE libraries. Therefore, the RNA-Seq comparison (now **Figure S16**) shows global TE expression normalized by global TE copy number in male and female genomes. We also expand our Method to estimate TE copy number in the genome for males and females using genomic DNA from embryos (which lack polytene chromosomes with under-replicated heterochromatin; **Figure S16B**), and directly from extracting TE copy number from the genome assembly (**Figure S16C**). We have also re-plotted the data in a more straightforward way by showing DNA-normalized TE expression for each sample (instead of male/female ratios).

A related question - the authors used *D. melanogaster* spike-in as a control to normalize between samples. While there is enough genetic divergence in unique sequences between the two species, it is unclear whether repetitive sequences also diverge enough to avoid cross-mapping. Could it be possible that D. melanogaster repeats from the spike-in confound the estimates for D. miranda?

We add **S1 Appendix** that describes rates of cross-mapping between *D. miranda* and *D. melanogaster*. We provide **Table A1** to show how many reads that originally mapped to *D. miranda* would map to either *D. miranda* or *D. melanogaster*. We show that, on average, about 0.6% of the reads aligned to *D. miranda* can cross-map to *D. melanogaster.* We did a similar approach using pure genomic DNA from *D. miranda*. We found that of 24,375,599 male reads mapped to *D. miranda*, 275,936 (0.1%) then mapped to *D. melanogaster* and of the 15,563,664 female reads mapped to *D. miranda*, 151,165(1%) mapped to *D. melanogaster*. These cross-mapping estimates are similar to those in the ChIP-data, suggesting that almost all reads mapped to *D. miranda* are likely derived from *D. miranda*.

 In **Table A2** we took reads originally mapped to *D. miranda* heterochromatin and mapped them to both *D. miranda* and *D. melanogaster* genomes to infer cross-mapping between the species’ repeats. We find that the rate is almost always less than 1% (especially when omitting Old Male Replicate 1) and this suggests low cross-mapping between species-specific repeats. Lower cross-mapping of heterochromatin between *D. miranda* and *D. melanogaster* presumably reflects the rapid turn-over of repetitive DNA between species (that is, repeats are typically less conserved between species than unique DNA).

2. One major finding from this study is that, compared to pericentromeric heterochromatin of other chromosomes, neo-Y has lower enrichment of H3K9me. Several aspects of this finding need to be further addressed.

- How non-unique sequences (not necessarily repetitive) were treated in the analysis? I presume that the neo-Y still has many shared sequences with the neo-X. A neo-Y ChIP-seq read could also map to neo-X, leading to averaging out the H3K9me3 enrichment level of estimated for neo-Y.

This is a good point, and we add **S2 Appendix** that evaluates cross-mapping between the neo-X and the neo-Y. The neo-Y is recently diverged from the neo-X and thus may potentially produce mis-mapping issues. We addressed this in a few different ways. First, we provide **Table B1** to show how many reads originally mapping to the neo-Y will map to either the neo-X or the neo-Y. We find that roughly 96% of reads originally mapped to the neo-Y will map back to the neo-Y while only 1% will map to the neo-X. These data suggest that cross-mapping between the two chromosomes is very low since the vast majority of reads will align back to the original chromosome.

Next, we simulated reads from the neo-X and the neo-Y using ART (Huang et al. 2012) and mapped both of these simulated read sets to the neo-X and the neo-Y. The following parameters were used to simulate 100-bp paired end reads (as were collected for our ChIP-seq experiment): -ss HS20 -l 100 -f 10 -p -m 375 -s 25. **Table B2** shows that overall, the vast majority of reads will map back to their respective chromosome and even more so when we apply an alignment quality cutoff. In particular, over 99% (or 99.9% of reads when using MAPQ>3 cut-off) of simulated neo-Y reads map back to the neo-Y chromosome, demonstrating that the neo-sex chromosomes have accumulated enough sequence differences for reads to map back uniquely to their respective chromosomes.

We then performed an analogous approach to infer mis-mapping from the neo-X to the neo-Y by using female genomic DNA from *D. miranda*. We mapped these DNA reads and took all reads mapping to the neo-X and re-mapped them to both the neo-X and the neo-Y. Of the original 2,042,127 neo-X alignments, 2,020,692 (98.9%) were aligned properly to the neo-X and the rest (1.1%) aligned to the neo-Y.  Similar to our results of mapping neo-Y reads to the neo-X, almost all reads will be reassigned to their initial chromosome (see **S2 Appendix**).

If mis-mapping were to occur from the neo-Y to the neo-X, one might expect that there would be elevated H3K9me3 levels on the neo-X in males compared to females. We provide **Review Figure 1** which shows the differences in H3K9me3 enrichment between the neo-X of females and males of similar ages. Overall H3K9me3 enrichment on the neo-X is the same between young males and young females (**Review Figure 1**). At neo-X heterochromatin, males consistently have less H3K9me3 enrichment than females (**Review Figure 1**).

**- Because of the uncertainty of how repetitive (#1) and non-unique (see above) sequences are treated in the reported ChIP-seq analysis, I feel it is necessary to use orthogonal methods to demonstrate that neo-Y indeed has lower enrichment of H3K9me3. A straightforward IF experiment should be able to address this.**

We provide **Figure 3B and Figure S10** showing an immunofluorescence stain done on male *D. miranda* (*MSH22*) using anti-H3K9me3 antibodies, following Fanti et al 2008. Please note that this was not a ‘straightforward’ experiment, but took weeks to optimize, with input from Laura Fanti and other colleagues. In our IF experiments, we see consistent and strong staining along the large (>20Mb) pericentromere of the metacentric X chromosome and pericentromeres at other chromosomes, and substantially weaker staining along the much longer, highly repetitive neo-Y chromosome. This supports our ChIP data, which show lower enrichment of H3K9me3 on the neo-Y.

- Authors suggested that the lower enrichment of H3K9m3 on the neo-Y could be due to the active transcription of genes on the neo-Y. This possibility could and should be further investigated, potentially with existing data. For example, authors can compare the H3K9me3 enrichment level in windows with different distances to transcribing genes, genes with different transcription levels etc, for both neo-Y and heterochromatin on other chromosomes.

This is a good point, and now more clearly discussed on p. 7. We provide a new figure **(Figure 4A)** showing H3K9me3 enrichment surrounding transcription start sites (TSS) in pericentromeres and on the neo-Y for young males. We find that the neo-Y has lower H3K9me3 at TSS compared to pericentromeres at other chromosomes and their TSS. Our TSS annotation of the D. miranda genome contains 6724 sites on the neo-Y and 327 sites in the pericentromeric regions.

To show that H3K9me3 enrichment could be due to active transcription, we also plotted the enrichment as a function of distance from highly expressed (top 20th percentile) of neo-Y genes in comparison to the lowly expressed (bottom 40th percentile) and all neo-Y genes (**Figure 4B**). We find that H3K9me3 enrichment is reduced around neo-Y genes with higher expression compared to genes with lower expression and also the global neo-Y enrichment. This supports our argument that active transcription of neo-Y genes interferes with heterochromatin

- A related and important question - it is intriguing how authors defined heterochromatin differs between neo-Y (using H3K9me3 gradient) and other chromosomes (using % of repeats). Heterochromatin is genomic regions enriched with H3K9me3. I am not sure why the authors chose other metrics for chromosomes other than the neo-Y. I also wonder whether some results could have been driven by this difference in methods between neo-Y and other heterochromatin.

True - different cutoffs are used to define regions as pericentromeric heterochromatin between the autosomes/neo-X and the neo-Y. We followed ref. [24] to define pericentromeric heterochromatin for the autosomes/neo-X based on repeat content (>40%). The neo-Y consists of, on average, 70% repeats along the entire chromosome (see Figure 1C), precluding us from using the same 40% cutoff as for the other chromosomes to identify pericentromeric heterochromatin. Thus, we used H3K9me3 enrichment to define the pericentromere region; note that this region also corresponds to the pericentromere when comparing homologous regions between the neo-X and the neo-Y chromosomes (see Figure 5A in Mahajan et al. (2018) PLoS Biology, 16(7): e2006348).

In **Figure S19**, we show where heterochromatin boundaries are called using the average female (young) ChIP sample. We show that regions to the left of the boundary overlap with elevated H3K9me3 signal. Thus, we don’t expect slight differences in the boundary of heterochromatin/euchromatin to affect our results.

3. Authors should include line numbers – it is hard to refer to specific passages without line numbers.

Done.

4. In order to know that authors' normalization methods work, it would be helpful to show some euchromatic loci/genes that are known to be stably enriched for K9. Especially, I am concerned about the cross-mapping of D. melanogaster sequences from the spike-in (#1).

We addressed the cross-mapping rates of *D. melanogasters* above and found that the rates for repetitive regions were low and similar to the rates from cross-mapping rates of pure species gDNA.

Because we do not know of euchromatic loci/genes that are known to be stably enriched with H3K9me3 in *D. miranda*, we instead used our data to show that the normalization methods were consistent in enrichment levels on a per-window basis. For each normalization method, we identified euchromatic regions whose H3K9me3 enrichment was in the top 20th percentile. We then compared these sets of regions and found that 99.13% of these regions are consistently identified in the top 20th percentile across all normalization methods (**Table S10**). These results suggest that regions enriched in H3K9me3 in the euchromatin are consistently enriched by the various methods.

A related question – Did the authors use orthologous euchromatic regions? Or any euchromatic regions? Only unique reads? If allowing any euchromatic regions and could be multi-mapped reads, I am worrying that the normalization could be influenced by the difference in H3K9me3 level on the neo-Y between samples (since neo-X and neo-Y still share some sequences, also see #2).

Our normalization methods do not use specific regions in the genome. The normalization is based on mappings to the entire *D. melanogaster* genome so they are not influenced by potential mis-mappings between the neo-X and neo-Y.

We explain our normalization methods better in the Methods of the paper. In our first approach, which underlies the main figures, we did not normalize the *D. miranda* H3K9me3 by the *D. melanogaster* spike-in. These data are normalized by library size for both ChIP and the inputs. For the next three methods (**Figure S1A/B**, tracks 2-4), we mapped the ChIP and input data simultaneously to also the entire *D. melanogaster* genome. In the second method (**Figure S1A/B**, track 2), we based the normalization by the total number of reads mapping to *D. miranda* and *D. melanogaster* in the different libraries, irrespective of where the reads map. This is similar to normalization methods of RNA-Seq data with a spike-in transcript.

In the last two methods (**Figure S1A/B**, tracks 3 and 4), we took the whole-genome alignments in *D. melanogaster* and generated ChIP enrichment plots for each sample’s spike-in. Then, we calculated a normalization factor by using all 5-kb sliding windows in the *D. melanogaster* genome, thus including enrichment from euchromatin and heterochromatin. Differences in H3K9me3 levels on the neo-Y/neo-X are not taken into account when calculating these normalizations.

Most importantly, our conclusions are robust with respect to normalization methods, and irrespective of whether we use the *D. melanogaster* spike-in or not.

5. I am confused with the authors' argument that active TE families are less epigenetically silenced, and this could explain the lower K9 enrichment on the neo-Y (page 6). At least in D. melanogaster, LTR families are recently active, targeted by a large amount of small RNAs, and more epigenetically silenced.

What we are discussing here is a theoretical possibility to explain less H3K9me3 enrichment on the neo-Y– that is, TE’s that are more abundant on the neo-Y may be TEs that have recently invaded the *D. miranda* genome, and may not yet have evolved efficient silencing mechanisms. TEs that recently invaded a new species may not yet produce small RNAs and thus show less heterochromatin enrichment. By comparing H3K9me3 enrichment of TEs from the same family on the neo-Y vs. other chromosomal locations, we show that TEs belonging to the same family have lower H3K9me3 when located on the neo-Y. We rephrase that paragraph to make it clear that we are talking about a hypothesis.

6. Authors need to explain why the epigenetic silencing of simple repeats and TEs in young and old males differ in *D. miranda* and *D. melanogaster*. The toxic Y hypothesis suggests that the source/sink relationship is the key, but does not distinguish between repeat types. This is central to the main theme of this study and needs to be discussed in depth.

We clarify this in the text, and add some more detailed discussions. In particular, we believe that the different genetic composition of the Y chromosome is a major reason of why the pattern of repeat expression differs between young and old *D. miranda* and *D. melanogaster*. The Y chromosome of *D. melanogaster* is an ‘old, degenerate” Y chromosome that only contains a handful of genes, and is efficiently epigenetically silenced in most tissues. The *D. miranda* neo-Y contains a mosaic of repetitive DNA and 1000s of genes that are widely expressed. Our data suggest that active transcription of neo-Y genes limits the formation of silencing heterochromatin on the neo-Y, and thus results in global up-regulation of TEs in male *D. miranda*. Unlike TEs, simple repeats are not enriched on the neo-Y of *D. miranda*, and are thus efficiently silenced in young males. This is now more clearly discussed in the paper in the Discussion.

Related to this - discussion on page 12 is confusing.

Authors started with that repeats on the Y lead to the toxic effect, but then stated that the differences between D. melanogaster and D. simulans are due to their difference in TE content on the Y. Authors failed to explain why the different TE content matters and whether the toxic Y theory is relevant only for TEs or both repeats.

We reworked that part of the paper, to make it less confusing. We state that the Y of *D. miranda* seems particularly toxic, for two reasons. On one hand, the neo-Y of *D. miranda* has more TE’s than *D. melanogaster’s* Y chromosome. In addition, those TEs on the neo-Y of *D. miranda* appear insufficiently silenced throughout adulthood, due to the presence of 1000s of actively transcribed genes on the neo-Y. The highly degenerated Y of *D. melanogaster* has much fewer genes and therefore TEs are efficiently silenced initially in adulthood, but silencing breaks down later in life. Presumably all repeats that are enriched on the Y should contribute to the toxic Y effect; however, satellites are not enriched on the neo-Y of *D. miranda*.

7. (Page 16) Authors excluded one old male sample (old male 1) based on the boxplot in Figure S1. However, the criterion for excluding that sample is unclear. By eyeballing, I feel that young female 2 is an outlier to other replicates and could also be excluded. Authors may consider more formal analysis (e.g., IDR) to include/exclude a replicate.

We provide **Table S3** showing the Pearson correlation coefficients between replicates of the same biological sample. A majority of the replicates within biological samples are consistent with each other with the exception of old male 1 (coefficient below 0.9). We also provide **Review Figure S2 s**howing a PCA plot of the replicates based on autosomes and X chromosome  and we note that old male 1 (“MO1”) splits farther away from the rest of the old males (“MO[2-4]”).

The IDR pipeline relies on peaks called from ChIP data (e.g. peaks from MACS2 or peakSeq) and we do not call peaks with our data; instead we calculated ChIP enrichment for genomic windows.

8. (Page 5) Authors reported that the majority of the windows that lost K9 are in pericentromeric heterochromatin or neo-Y. However, these genomic regions are also enriched with K9-enriched windows. It could be that the whole genome is losing K9. A more appropriate analysis would be to compare the proportion of windows in each genomic compartment (Het, neo-Y, Eu) that lost K9.

In **Figure S9**, we show the proportion of windows that exhibit at least a 1.5-fold change difference in H3K9me3 over aging in males and females. We find that few regions in euchromatin exhibit a loss of greater than 1.5x in comparison to the number of regions in heterochromatin and, to a lesser extent, the neo-Y.

Related to this - it is intriguing that the number of windows with increased K9 is also more for males than females. I wonder whether this has to do with the uneven coverage between males and females (assuming sequencing depth is the same, the average coverage would be higher for females due to the overall smaller genome size). This could lead to a larger variance for estimates in males. This could potentially be addressed by downsampling female data to the same genome coverage.

We note that females have a larger genome size because they have 2 copies of both the old-X (MullerA-AD) and the neo-X (Muller C) in contrast to males. However, the female genome assembly is 35% smaller than the male assembly (173.1Mb vs 265.7Mb). We downsampled the female data to the same genomic coverage of the male data (35% less reads than male data).

We provide **Review Figure 3** which shows a figure similar to **Figure S9** but with female data downsampled. In males 797 windows show increased H3K9me3 over aging in comparison to 202 windows for females suggesting that this increase is not necessarily due to variation in the sex-specific assembly size. This is consistent with our notion that H3K9me3 is redistributed over aging.

9. Authors mentioned that male-male aggression could also lead to lower survival of the males. Since the authors mentioned it, it would be helpful to provide either a rationale why this did not impact the authors' results or some empirical validation (e.g., rear flies individually to estimate the lifespan).

Over 3000 flies were assayed for lifespan, thus it is not feasible to rear them individually to estimate lifespan. Males were kept in cohorts of 30 as described in [Linford 2013], as is typically done for aging studies in the field.

10. In Figure S13, no p-values were provided (neither in the text). By looking at the figure, there are no obvious differences in TE expression between males and females (which contradicts with authors' claim on page 9).

We want to clarify that our original **Figure S13** showed Log2(male)-Log2(female) TE expression and this is equivalent to Log2(male/female). Anything above the grey line indicated higher expression in males than females. Each boxplot represented fold-changes in expression for different ages and for values that are, or are not, normalized by genomic TE copy number. All of the boxplots were above this grey line and this indicated that males have higher TE expression than females irrespective of age and whether TE copy number is taken into account.

We reworked this figure to show normalized expression for each sample instead of ratios of samples (**Figure S16 A-C**). We also present scatterplots **(Figure S16 D-F)** which show that high TE expression in males is irrespective of TE copies. P-values from two-sided, unpaired Wilcoxon test (* = p < 0.05, n.s. = not significant).

11. I am not sure what H3K9me3 Enrichment "Signal" refers to in Figure 2 and some other figures. I think the authors meant "H3K9me3 Fold Enrichment"?

Yes, we label our ChIP/Input data as “H3K9me3 Enrichment Signal,” which is the same as ChIP/Input fold-enrichment.

12. Some of the numberings of supplementary figures are wrong (e.g., Figure S11 on page 9, some of those should be Figure S12).

Thanks – we made sure that the supplementary figures are numbered correctly in our revised version.

**Response to Reviewer #2:**

This is a very nice manuscript describing sex-specific derepression of transposable elements with age in a Drosophila species with a large Y chromosome. The manuscript is well-written and clear, though I would like to see some minor points addressed.

An alternative to the toxic-Y explanation for some of the results is a male-specific decline in proteins required for heterochromatin formation. I'd agree that this is less likely, but perhaps they can address this alternative with a few more analyses of the RNAseq and ChIPseq data already collected-- e.g., is there a decline in expression in males with age of any relevant genes that is strong relative to that in females? Are overall H3K9me3 levels similar between males and females of the same age? This may be particularly important to show for X-linked genes, particularly as the neo-X in this group is, as I recall, not fully dosage compensated.

This is a good point, and we provide additional figures leveraging our existing RNAseq data, and this is now briefly mentioned in the paper (p. 5 and **Table S6**).

**Reviewer Figure 4** shows volcano plots of genes for comparisons within sexes (A: males, B: females, left: higher expression in old samples, right: high expression in young samples) and between sexes (C, D ignoring neo-Y genes; left: higher expression in females, right: higher expression in males).

We looked at the fold-changes between young and old samples of 34 genes involved in nuclear heterochromatin, heterochromatin assembly, and heterochromatin organization from Flybase (**Table S6**). In males, 14/20 of these genes are down/up-regulated during aging, and 12/22 in females. The differences in expression between old and young samples is small (median Log2-fold change is 0.16 in both males and females), and we find only two genes whose expression is significantly different between young and old males (*Atf-2* and *Arp6*).  *Atf-2* (*Activating transcription factor 2*) is required for heterochromatin assembly and also involved in stress response while *Arp6* (*Actin related protein 6*) is believed to be involved with histone exchange. Thus, these data suggest that almost all heterochromatin-associated genes are expressed stably during aging in flies, and there are only minor differences between the sexes.

We include **Reviewer Figure 1** which shows overall H3K9me3 levels compared between males and females of the same age separated by autosomes (A) and neo-X (B). We find that *overall* H3K9me3 are similar in both males and females of the same age. In **Reviewer Figure 1,** we made our comparison of overall H3K9me3 levels at pericentromeric regions more explicit.

While I appreciate that the authors focussed on well-founded conclusions, I did feel they could speculate a bit more about the proximate causes of the hypothesized toxic Y-effect. e.g., TE activity leading to DNA damage and cell-death in the soma. On a related point, there is something of a lack of non-fly literature on this topic cited (e.g.,de Cecco et al Nature volume 566, pages73–78(2019)), where some of these details have been hammered out.

Similarly, I was surprised to see no mention of the essentially similar work of Lemos et al (e.g., PNAS September 7, 2010 107 (36) 15826-15831)

This is a good point and we now include a discussion of possible proximate causes of toxic Y effect in the discussion. We now also cite these two references in the paper.

Finally, I am a wondering how well the attempt to correct expression for TE copy numbers works. The standardisation used, as I understand it, is to divide RNAseq reads by DNA reads. While logical, it is worth remembering that heterochromatin is underreplicated, so that TE copies that are effectively heterochromatin-ised by be underrepresented in the DNA data, and therefore undercounted. I don't think this materially affects their conclusions, but would appreciate an argument showing this, as it is effected by exactly the process they are trying to measure. Or, alternatively, they could correct with early embryo DNAseq data if any exists. (As heterochromatin does not yet form in early embryos.)

We acknowledge that the gDNA libraries from which the TE copies are calculated have an underrepresentation of heterochromatin. We expand **Figure S16**, and now also show normalized expression when TE copy number is calculated from early embryo genomic DNA (developmental Stage 3; **Figure S16B**) and by using a repeat annotation of genome assemblies for both sexes (**Figure S16C**). Both figures show that males have higher TE expression than females when accounting for these newly calculated copy numbers.

p8-- the word 'access' is used, but I think 'excess' is meant.

We fixed this typo.
